# Identification, heterologous production and bioactivity of lentinulin A and dendrothelin A, two natural variants of backbone N-methylated peptide macrocycle omphalotin A

**DOI:** 10.1038/s41598-021-83106-2

**Published:** 2021-02-11

**Authors:** Emmanuel Matabaro, Hannelore Kaspar, Paul Dahlin, Daniel L. V. Bader, Claudia E. Murar, Florian Staubli, Christopher M. Field, Jeffrey W. Bode, Markus Künzler

**Affiliations:** 1 Department of Biology, Institute of Microbiology, ETH Zürich, Room HCI F409, Vladimir-Prelog-Weg 4, CH-8093 Zürich, Switzerland; 2 Agroscope, Phytopathology and Zoology in Fruit and Vegetable Production, Müller-Thurgau-Strasse 29, CH-8820 Wädenswil, Switzerland; 3 Department of Chemistry and Applied Biosciences, Laboratorium Für Organische Chemie, ETH-Zürich, Vladimir-Prelog-Weg 3, CH-8093 Zürich, Switzerland; 4 Institute of Transformative Bio-Molecules (WPI-ITbM), Nagoya University, Chikusa, Nagoya 464-8602 Japan

**Keywords:** Environmental biotechnology, Natural product synthesis

## Abstract

Backbone N-methylation and macrocyclization improve the pharmacological properties of peptides by enhancing their proteolytic stability, membrane permeability and target selectivity. Borosins are backbone N-methylated peptide macrocycles derived from a precursor protein which contains a peptide α-N-methyltransferase domain autocatalytically modifying the core peptide located at its C-terminus. Founding members of borosins are the omphalotins from the mushroom *Omphalotus olearius* (omphalotins A-I) with nine out of 12 *L*-amino acids being backbone N-methylated. The omphalotin biosynthetic gene cluster codes for the precursor protein OphMA, the protease prolyloligopeptidase OphP and other proteins that are likely to be involved in other post-translational modifications of the peptide. Mining of available fungal genome sequences revealed the existence of highly homologous gene clusters in the basidiomycetes *Lentinula edodes* and *Dendrothele bispora*. The respective borosins, referred to as lentinulins and dendrothelins are naturally produced by *L. edodes* and *D. bispora* as shown by analysis of respective mycelial extracts. We produced all three homologous peptide natural products by coexpression of OphMA hybrid proteins and OphP in the yeast *Pichia pastoris*. The recombinant peptides differ in their nematotoxic activity against the plant pathogen *Meloidogyne incognita*. Our findings pave the way for the production of borosin peptide natural products and their potential application as novel biopharmaceuticals and biopesticides.

## Introduction

Fungi constitute a treasure trove of diverse bioactive natural products, many of which are used as valuable therapeutics to treat infectious diseases or cancer, or as immunomodulatory agents^1,2^. Recent discoveries of novel fungal peptide natural products and their biosynthetic pathways have raised particular interest in these compounds^3^. Peptide natural products include non-ribosomal peptides (NRPs) and ribosomally synthesized and post-translationally modified peptides (RiPPs). NRPs are made by huge multimodular enzymes called non-ribosomal peptide synthetases (NRPSs)^4^. These enzymes are able to assemble peptides from proteinogenic and non-proteinogenic amino acids independently of the ribosome. Some of these assembly lines also contain modules that chemically modify the building blocks, such as α-N-methyltransferases^4–6^. RiPPs, on the other hand, are derived from ribosomally synthesized precursor peptides or proteins^7^. The precursor protein usually undergoes posttranslational modification (PTM), followed by proteolytic cleavage of the core peptide from the precursor protein. Typical PTMs include cyclization, N-methylation (of both backbone and side chain nitrogens) and introduction of thioether linkages^8,9^. Modifications of both NRPs and RiPPs significantly impact the pharmacokinetics of the respective peptide natural products. The peptides become more membrane permeable, are more resistant to proteolytic degradation and display improved target specificity, all of which enhances their applicability as therapeutic agents^10–13^.

**Figure 1:**
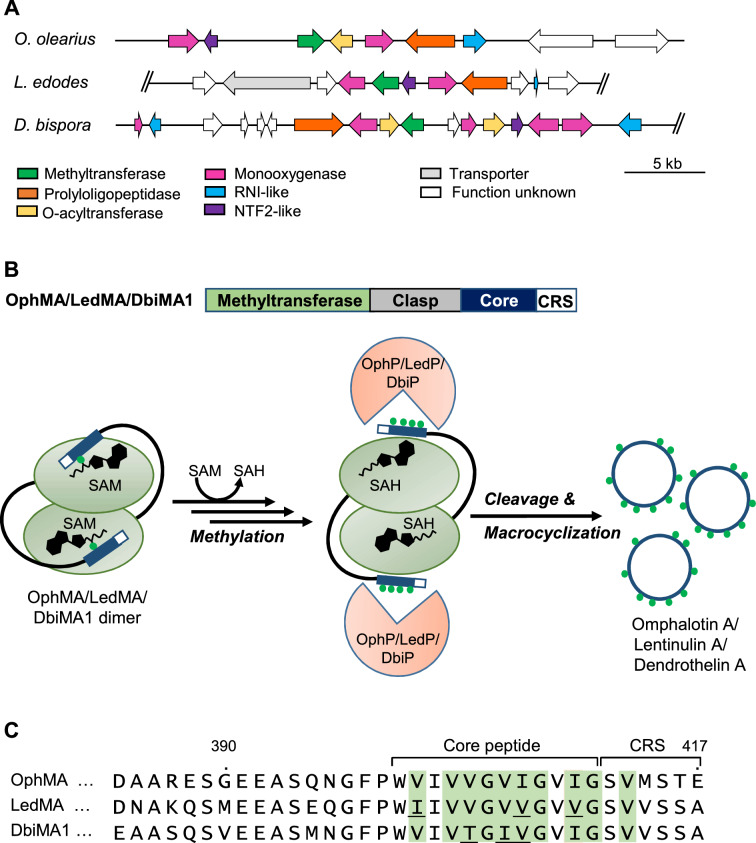
Structure and function of omphalotin biosynthetic gene cluster and homologous clusters in the basidiomycetes *O. olearius*, *D. bispora* and *L. edodes*. (**A**) Schematic representation of the borosin biosynthetic gene clusters in the basidiomycetes *O. olearius* (VT 653.13, scaffold_169), *L. edodes* (Le(Bin) 0899 ss11, scaffold_10) and *D. bispora* (CBS 962.96, scaffold_621). All three clusters code for a precursor (methyltransferase) protein, referred to as OphMA, LedMA and DbiMA1 respectively, and a prolyloligopeptidase, referred to as OphP, LedP and DbiP, respectively. The DNA scaffolds and filtered gene models were taken from https://mycocosm.jgi.doe.gov/. Double slashes (//) indicate that the sequence of the DNA scaffold continues beyond this position. (**B**) Biosynthesis scheme of omphalotin A and closely related peptides, lentinulin A and dendrothelin A as a combined action of the precursor (methyltransferase) protein and the prolyloligopeptidase. (**C**) Alignment of the C-termini of the precursor (methyltransferase) proteins from *O. olearius* (OphMA), *L. edodes* (LedMA) and *D. bispora* (DbiMA1). Residues differing from the omphalotin core peptide are shown in red. The indicated methylation patterns (green fill) were previously determined by heterologous expression of the respective cDNAs in *E. coli*^21,23,25^. CRS = C-terminal recognition sequence.

Until recently, backbone N-methylation had only been reported for non-ribosomal peptides like cyclosporine A, a fungal undecapeptide natural product used as an immunosuppressant in organ transplantation^13^. Advances in genome sequencing technologies^14^ and mass spectrometry (MS)-based peptide detection^15^ led to the recent discovery of several novel fungal RiPP classes, including the backbone-N-methylated borosins^3^. Founding members of the borosins are the omphalotins from the mushroom *Omphalotus olearius* (omphalotins A-I), with nine out of 12 *L*-amino acids being backbone N-methylated^16^. These macrocyclic peptides had been discovered, more than twenty years ago, based on their toxicity against nematodes, in particular towards *Meloidogyne incognita*, an agricultural important plant pathogen^17–19^. Mining of the *O. olearius* genome revealed the omphalotin biosynthetic gene cluster encoding, besides a number of putative omphalotin modifying enzymes, the omphalotin precursor protein OphMA and the prolyloligopeptidase OphP (Fig. 1A)^20,21^: OphMA is composed of a SAM-dependent methyltransferase domain which methylates multiple residues of its C-terminal core peptide region^22^, while OphP excises and catalyzes the macrocyclization of the methylated core peptide of OphMA^20^ (Fig. 1B). In the meantime, OphMA homologues were identified in several fungal genomes suggesting a large diversity of yet unexplored members of the borosin class of backbone N-methylated RiPPs^23^. Apart from gymnopeptides^24^, none of these peptide natural products have been identified and characterized, however.

Interestingly, gene clusters with high similarity, both in composition and organization, to the omphalotin biosynthetic gene cluster have been found in other agaricomycetes notably the edible mushrooms *Lentinula edodes (Shiitake),* which belongs to the same family of Marasmiaceae as *O. olearius,* and *Dendrothele bispora* belonging to the family of Corticiaceae^20,21,23,25^*.* Genes shared by these clusters include those coding for OphMA, OphP and other enzymes such as monooxygenases and O-acyltransferases likely responsible for additional modifications of the peptide natural product (Fig. 1A). Inspired by these observations and a previous report on the heterologous production of omphalotin A in *Pichia pastoris*^20^, we set out to produce the predicted peptide natural products of these two fungi in this heterologous host. We first reproduced the previous results and produced omphalotin A by coexpressing OphP and OphMA*.* Analogously, we produced the predicted borosins from *L. edodes* and *D. bispora* by expressing OphP together with OphMA hybrids in which the core peptide region of OphMA was replaced by the one of the respective OphMA homologues. The two produced macrocyclic dodecapeptides were termed lentinulin A and dendrothelin A, after *Lentinula* sp. and *Dendrothele* sp., respectively, and displayed nine backbone N-methylations in the same pattern as in omphalotin A. This result is in accordance with the previously reported methylation pattern of the respective precursor proteins in *E. coli*^21,23,25^. We further confirmed that lentinulins and dendrothelins are naturally produced by *L. edodes* and *D. bispora* as shown by mass spectrometric analysis of respective vegetative mycelial extracts. Similarly to the omphalotins, we assume that these peptides are used as defense effectors against fungivorous nematodes and other antagonists. Thus far, the three recombinant macrocycles were tested against the plant-parasitic nematode *M. incognita,* where all of the compounds exhibited nematotoxic activity. These results show that *P. pastoris* represents a useful platform for the discovery and characterization of fungal borosin natural products. The platform may also be utilized for the generation of new-to-nature backbone N-methylated peptides with interesting biological activities^3^.

## Results and discussion

### Production and functional analysis of the OphMA (methyltransferase) precursor protein and hybrids thereof in *P. pastoris*

**Figure 2:**
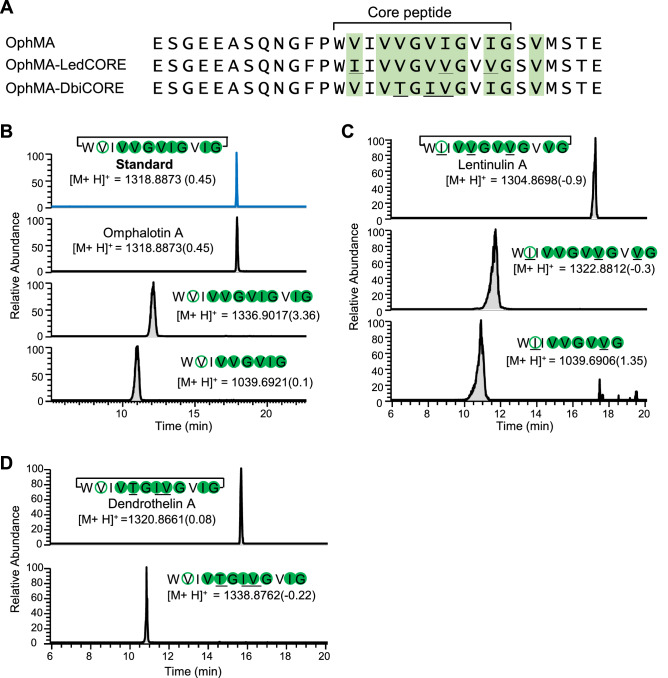
Production of backbone N-methylated peptide macrocycles in *P. pastoris*. (**A**) Methylation pattern of OphMA, OphMA_LedCORE and OphMA_DbiCORE. Methylated residues were determined by LC–MS/MS analysis of the C-terminal tryptic fragments and are shaded in green (Supplementary Fig. S2A–C). (**B**) Production of omphalotin A. The extracted compound was detected using LC–MS and compared to the chemically synthesized omphalotin A standard (216 ng/ml). In addition to omphalotin A, fully methylated, linear (core) peptides and short linear peptides lacking the C-terminal three residues (VIG) were detected. (**C**) Production of lentinulin A and (**D**) dendrothelin A. The detected peptides were confirmed by LC–MS/MS. The confirmed positions of methylation are depicted using filled circles, while methylated residues inferred from MS/MS are shown in open circles. Residues different from omphalotin A are underlined. The mass difference (represented in ppm) between observed values and theoretical mass is indicated in brackets for each compound.

The previously cloned cDNA of *ophMA* from *O. olearius*^21^ was recloned on the pPICZA vector under control of the *AOX1*-promoter and the recombinant plasmid was integrated at this locus in the genome of *Pichia pastoris* strain GS115. In order to produce the OphMA protein, the respective *P. pastoris* transformant was cultivated for three days at 30 °C in buffered methanol-containing complex medium (BMMY) and the N-terminally His8-tagged protein was purified over Ni–NTA beads. The methylation pattern of the purified protein was determined by tryptic digestion followed by LC–MS/MS analysis of the C-terminal tryptic fragment. The pattern was found to be the same as for OphMA produced in *Escherichia coli*, with the tenfold methylated species (9 methylations in the core peptide plus 1 methylation in the C-terminal recognition sequence) being the most abundant one (Figs. 1C and 2A)^21^.

Despite high levels of expression of *ledMA* cDNA in *E. coli*^23^, expression of the same sequence in *P. pastoris* yielded very low amounts of LedMA protein. Therefore, we constructed *ophMA* hybrid genes where the coding information for the OphMA core peptide was exchanged to the ones of the homologous genes *ledMA* and *dbiMA1* (also referred to as *dbophMA*^22,25^) coding for hybrid precursor proteins OphMA_LedCORE and OphMA_DbiCORE*,* respectively (Fig. 2A). The hybrid proteins were produced and analyzed as described above for the OphMA protein (Supplementary Fig. S1B). The methylation pattern of the purified hybrid proteins was identical to the one of OphMA, with the most abundant species featuring ten methylations, of which one methylated valine residue was located in the C-terminal recognition sequence (Fig. 2A and Supplementary Fig. S1, panels C–E). These results are in line with previous reports that showed that OphMA is relatively promiscuous with regard to the core peptide sequence^26^. In fact, van der Velden and colleagues^21^ demonstrated that OphMA is able to methylate sequences that are completely unrelated to the sequence of the omphalotin core peptide. Furthermore, expression of *ledMA* and *dbiMA1* in *E. coli* in the course of the functional and structural characterization of the respective precursor proteins yielded the same methylation pattern highlighting the functional similarity between all three enzymes^21,23,25^.

### Production of Omphalotin A and homologous peptides in *P. pastoris*

Given the production of fully methylated OphMA-derived precursor proteins in the yeast *P. pastoris*, we set out to produce the respective peptides by coexpression of *ophP* in these cells. For this purpose, *ophP* cDNA from *O. olearius* was inserted into the pPIC3.5 K vector under control of the *AOX1*-promoter and the recombinant plasmid was integrated at the *HIS4*-locus of *P. pastoris* strain GS115. In order to increase the solubility of OphP, a Strep-SUMO*-tag was fused to the N-terminus of the protein via a TEV cleavage site. An OphP-producing transformant (GS115-OphP) was selected and first transformed with the pPICZA vector carrying the *ophMA* gene targeted at the *AOX1* locus. The resulting double transformant (GS115-OphP-OphMA) was analyzed for coexpression of OphP and OphMA (Supplementary Fig. S2). Double transformants carrying pPICZA vectors directing the expression of OphMA_LedCORE (GS115-OphP-OphMA*_*LedCORE) or OphMA_DbiCORE (GS115-OphP-OphMA*_*DbiCORE) were analogously generated and subsequently analyzed. The presence of peptides in the cell extracts or culture media of the double transformants was assessed by organic extraction and subsequent analysis by liquid chromatography (LC)-coupled mass-spectrometry (MS). Omphalotin A was first chemically synthesized and used as standard for the MS detection of the expected backbone N-methylated peptides produced by the *P. pastoris* transformants. The synthesis of the very hydrophobic macrocyclic peptide was performed by established Fmoc manual solid phase peptide synthesis, followed by standard resin cleavage, cyclization reaction and several purification steps (see Supplementary Information for details). This procedure yielded around 6.5 mg of pure omphalotin A.

**Figure 3:**
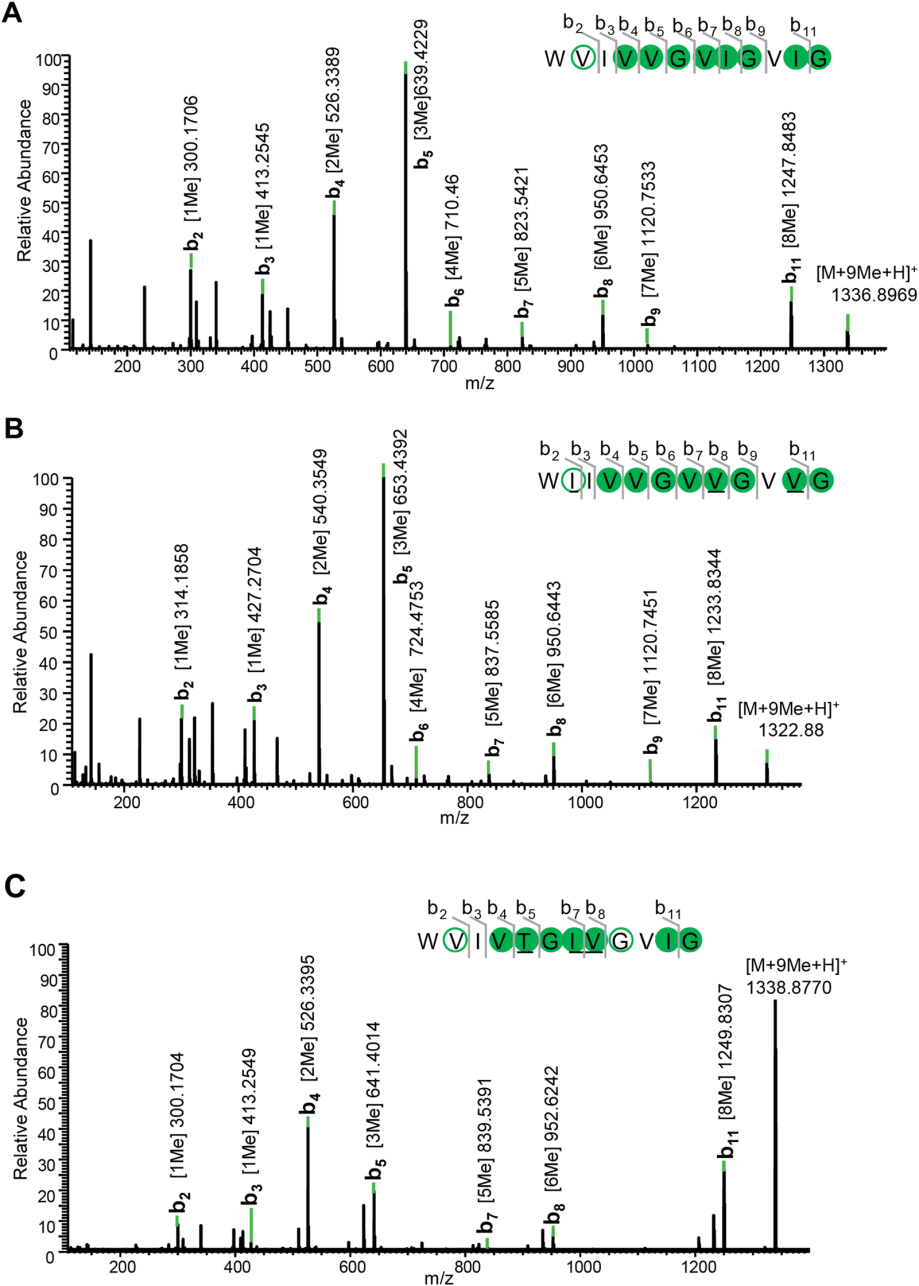
Annotated LC–MS/MS spectra of fully methylated, linear core peptides of omphalotin A, lentinulin A and dendrothelin A produced in *P. pastoris*. The peptide sequence of each peptide is presented together with the corresponding b-ions. The confirmed positions of methylation are depicted using filled circles, while methylated residues inferred from MS are shown in open circles. Residues different from omphalotin A are underlined. Annotated LC–MS/MS spectra of (macrocyclic) omphalotin A, lentinulin A and dendrothelin A are provided in Supplementary Fig. S5.

For the biosynthesis of omphalotin A, *P. pastoris* transformant GS115-OphP-OphMA was cultivated for three days at 30 °C in BMMY, as for the production of the biosynthetic enzymes. The yeast cells were lysed with glass beads and the peptides were extracted from the cleared cell lysate by phase separation using ethyl acetate or a mixture of ethyl acetate and n-hexane (1:1, v/v). The organic phase was collected and evaporated. The resulting pellets were dissolved in methanol and analysed by LC–MS/MS. The extracted ion chromatogram (EIC) of the *P. pastoris* extract yielded a peak at a retention time of 17.89 min, which was identical to the one of the chemically synthesized omphalotin A standard (M + H^+^ = 1318.89 Da). In addition to cyclic omphalotin A, a previously reported linear version of the ninefold methylated peptide (M + H^+^ = 1336.89 Da) was detected in the organic extracts of the *P. pastoris* cleared lysates^27^ (Figs. 2B and 3A). Unexpectedly, we also detected short linear peptides corresponding to the first nine amino acids (M + H^+^ = 1039.69 Da) of the core peptide carrying seven methylations (Fig. 2B and Supplementary Fig. S3A). These short linear peptide species were also observed in analogous extracts of *O. olearius* vegetative mycelium (Supplementary Fig. S6A). All peptides were confirmed by high-energy collisional dissociation tandem-MS (HCD-MS/MS) (Fig. 3A and Supplementary Fig. S4A).

Our results confirm a previous report demonstrating that OphMA and OphP are sufficient to produce omphalotin A^20^, and prompted us to produce the putative peptide natural products from the other two basidiomycete species using the respective OphMA hybrids in *P. pastoris*. For the production and detection of these peptides, we used the same procedure as for omphalotin A. The peptides were extracted from cleared cell lysates by phase separation using ethyl acetate. The organic phase was collected and evaporated, and the pellet was dissolved in methanol and analysed by LC–MS/MS. In the case of GS115-OphP-OphMA*_*LedCORE, the production of the ninefold methylated macrocyclic lentinulin A (M + H^+^  = 1304.87 Da) and the linear species thereof (M + H^+^  = 1322.88 Da) was confirmed by EIC peaks at retention times of 17.20 min and 11.9 min, respectively (Fig. 2C). Similar to omphalotin A, short linear peptides corresponding to the first nine N-terminal amino acids of the lentinulin A core peptide carrying seven methylations were detected (Fig. 2C and Supplementary Fig. S3B). Similarly, the production of macrocyclic dendrothelin A with ninefold methylation (M + H^+^ = 1320.86 Da), along with the full-length linear peptide species (M + H^+^  = 1338.87 Da) was confirmed for GS115/OphP-OphMA*_*DbiCORE, with EIC retention times of 17.20 min and 11.3 min, respectively, (Fig. 2D, and Supplementary Fig. S3C). In contrast to omphalotins and lentinulins, we could not detect any short linear dendrothelins. In all cases, the identity of the detected peptides was confirmed by LC–MS/MS (Fig. 3, panels B and C, and Supplementary Fig. S4, panels C–E). Based on the high degree of methylation of all macrocyclic and linear peptides produced, we hypothesize the existence of a checkpoint for OphP action to avoid premature cleavage of OphMA and ensure complete methylation of the peptide natural product. The nature of this checkpoint is unclear but it must be intrinsic to the bipartite OphMA/OphP system. Eventually, the recognition site(s) for OphP on OphMA is (are) only accessible upon a certain degree of backbone N-methylation of the core peptide (indirect recognition), or OphP recognizes the number of methylations in the core peptide of OphMA (direct recognition).

In order to assess the recognition of the omphalotin-related precursor proteins by the respective prolyloligopeptidases, we tested the cross-reactivity of these proteins. Since the prolyloligopeptidase of *D. bispora* (DbiP) predicted by the DOE Joint Genome Institute (https://mycocosm.jgi.doe.gov/) lacks the N-terminus and *ledMA* turned out to be difficult to express in *P. pastoris*, we only tested the cross-reactivity of the *L. edodes* prolyloligopeptidease (LedP) towards OphMA and hybrids thereof. In a first experiment, the transformant GS115-OphMA producing OphMA was transformed with a pPIC3.5 vector carrying *ledP* targeted to the *HIS4*-locus and the above protocol for production and analysis of peptides in *P. pastoris* was applied. The detected amounts of Omphalotin A, both in cells and in the culture supernatant of GS115-OphMA-LedP double transformants, were comparable to GS115-OphMA-OphP double transformants or even a bit higher (Supplementary Fig. S5A). In a second series of experiments, we assessed whether the two prolyloligopeptidases differentiate between the core peptide and CRS sequences of the precursor proteins. For this purpose, we tested the in vivo activity of OphP and LedP on above described OphMA hybrid proteins in which we exchanged the core peptide sequences. In order to address the role of the CRS, another OphMA hybrid protein, OphMA_DbiCterm, was produced. In this construct, we exchanged the core peptide plus CRS of OphMA by the respective C-terminus of DbiMA, in order to compare it to OphMA_DbiCORE with regard to processing by OphP (and LedP). We did not produce an OphMA_LedCterm hybrid protein as the CRS of LedMA and DbiMA are identical (SVVSSA), but different from the CRS of OphMA (SVMSTE) (Fig. 1C). All hybrid genes were coexpressed in *P. pastoris* transformants already producing either OphP or LedP, using the conditions and methods described above. In all cases, we obtained the respective macrocyclic peptides and the linear species thereof, some of which with lower degree of methylation, albeit with different ratios for the two prolyloligopeptidases (Supplementary Fig. S5, panels B and C). These results suggest that the two tested prolyloligopeptidases, OphP and LedP, are promiscuous for the core peptides or other regions of the precursor proteins, but LedP is slightly more active than OphP in *P. pastoris*.

### In vitro nematotoxicity of recombinant backbone N-methylated macrocycles against the plant pathogen *M. incognita*

Omphalotins produced from fermentation of *O. olearius* were reported to have strong and highly specific nematotoxic activities against the plant pathogen *M. incognita*^16,18,19^. Thus, we wanted to know if also recombinant omphalotin A and the homologous peptides are biologically active against *M. incognita*^28^. To do this, the peptides extracted from *P. pastoris* cells by phase separation were further purified using a C18 column on a preparative reversed-phase high performance liquid chromatography (RP-HPLC) system (Supplementary Fig. S7). In this way, 1200 µg, 800 µg and 400 µg of at least 95% pure peptide were obtained for omphalotin A, lentinulin A and dendrothelin A, respectively. The nematotoxicity of these compounds was assessed based on exposure of second-stage juveniles (J2) of the nematode for 1, 3, 7 and 14 days at peptide concentrations of 0.1, 0.75, 1.5 and 3 µM. The results revealed a strong nematotoxicity of both omphalotin A and lentinulin A at day 1 with lethal concentrations 50% (LC_50_) of 0.840 μM and 0.341 μM, respectively. The nematotoxicity of dendrothelin A was significantly lower with no detectable toxicity at day 1 and 3, and LC_50_ values of 0.472/0.167 μM at day 7/day 14 compared to 0.125/0.067 μM and 0.093/0.063 μM for omphalotin A and lentinulin A, respectively, after the same time period (Table 1, Supplementary Fig. S8 and Supplementary Dataset 1).

**Table 1:** In vitro nematotoxicity of recombinant omphalotin A, lentinulin A and dendrothelin A towards *M. incognita.*

	Day 1		Day 3		Day 7		Day 14	
	LC50	95% CI	LC50	95% CI	LC50	95% CI	LC50	95% CI
Lentinulin A	0.341	0.308	0.121*	<< 0.1	0.093	0.072	0.063	0.047
		0.377		>> 3.0		0.121		0.083
Dendrothelin A	>> 3.0*	6.173	>> 3.0*	<< 0.1	0.472	0.371	0.167	0.120
		>> 3.0		>> 3.0		0.601		0.231
Omphalotin A	0.840	0.772	0.245	0.212	0.125	0.094	0.067	0.047
		0.914		0.283		0.166		0.095
Fluopyram	0.375	0.337	0.084*	<< 0.1	0.040	0.029	0.060*	<< 0.1
		0.417		>> 3.0		0.056		>> 3.0

The nematotoxicity of the three macrocycles was determined at different concentrations and exposure times on *M. incognita* second-stage juveniles (J2) as described in Materials and methods. Shown are the estimated lethal concentrations 50% (LC50) in μM and the 95% confidence interval (95% CI) on those estimates. * indicates that the statistical model did not adequately fit the data and thus that the values are less confident (see Supplementary Dataset 1 for raw data and Supplementary Fig. S8 for statistical analysis). The nematicide fluopyram was used as positive control.

The nematotoxic activities of recombinant omphalotin A and lentinulin A towards *M. incognita* after 24 h (1 day) are comparable to the previously reported activity of omphalotin A^16^ (LC_50_ of 0.57 µM after 16 h) and of omphalotins E-I^18,29^ (LC_50_ between 0.38 and 3.8 μM) purified from *O. olearius*. According to these previous studies, the viability of the bacterivorous model nematode *Caenorhabditis elegans* and other plant parasitic nematodes such as *Heterodera schachtii*, *Radopholus similis* and *Pratylenchus penetrans* was also affected by omphalotin A, however, at a significantly higher concentration than *M. incognita*^16,18,29^*.* At the moment, the basis of the apparent specificity of omphalotin A towards *M. incognita* is unclear. It may be caused by differences in the presence, the structure or accessibility of the targets or in the stability of the peptide in the different nematodes. Given the comparable activity of omphalotin A and lentinulin A and the reduced activity of dendrothelin A, it can be concluded that the Val residue mutated to Thr in dendrothelin A, is important for the activity, at least towards *M. incognita*. Our platform may be used to obtain further insight into this issue by producing additional, new-to-nature variants of the peptide and test them for activity against a panel of different nematodes.

### Natural production of Lentinulins and Dendrothelins in vegetative mycelia of the respective mushroom species

The discovery of gene clusters homologous to the omphalotin biosynthetic gene cluster in *D. bispora* and *L. edodes* suggests that these mushrooms produce peptide natural products similar to the omphalotins. Accordingly, Quijano et al. (2019) confirmed the expression of *ledMA* in *L. edodes* vegetative mycelium, but not in the fruiting body by RT-PCR^23^. Given these results and the successful production of the predicted peptides in *P. pastoris*, we set out to confirm the production of the predicted peptides in *D. bispora* and *L. edodes* using *O. olearius* as control. Since natural product biosynthesis is usually tightly regulated and dependent on cultivation conditions and host organism^30^, we cultivated vegetative mycelium of all three fungi under two different conditions, on agar-solidified and in liquid YMG medium. For all cultures, the presence of the peptides was assessed, as described above for *P. pastoris*, both for fungal cell lysates and culture supernatants (in case of liquid cultures).

**Figure 4:**
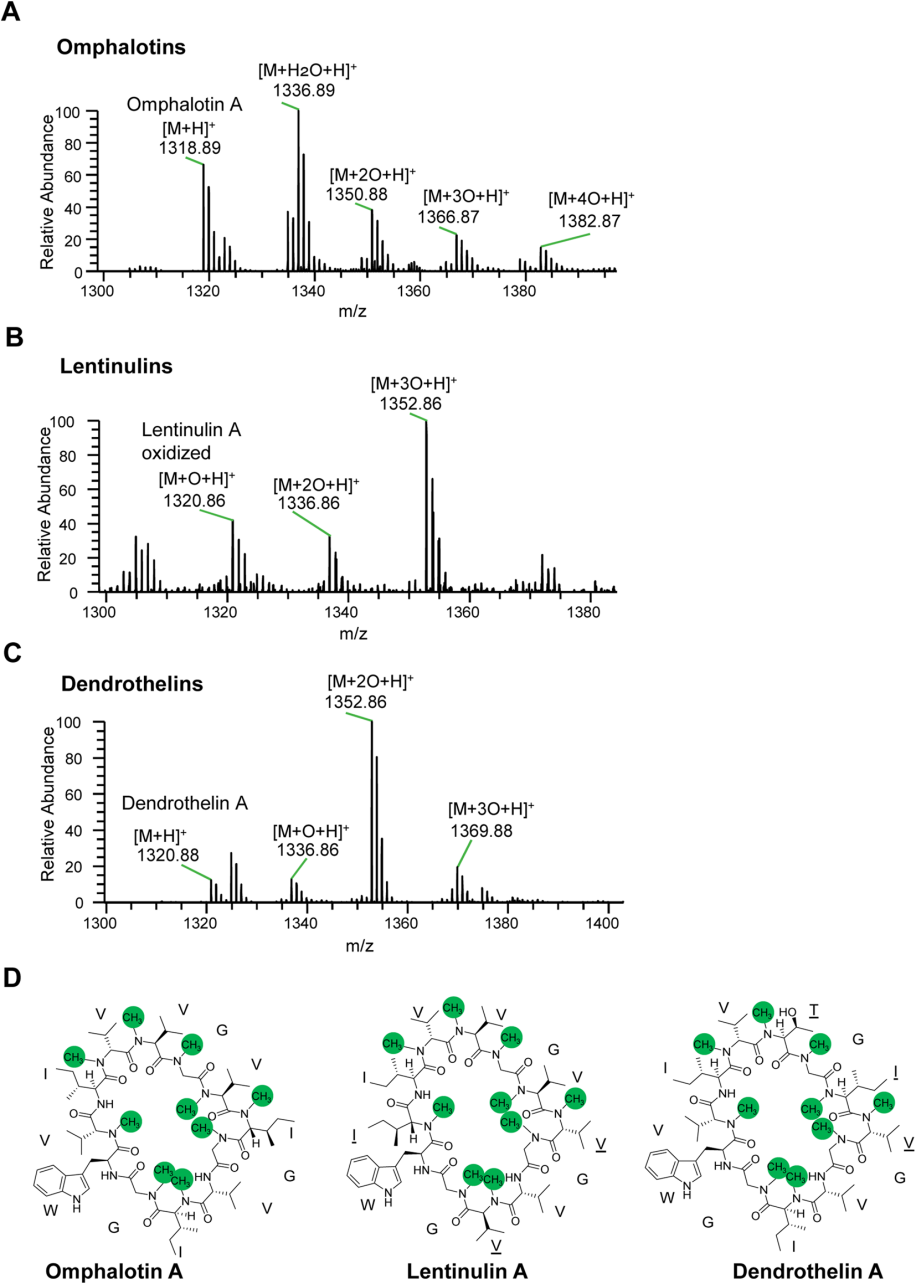
LC–MS analysis of backbone N-methylated peptides extracted from mushrooms *O. olearius*, *L. edodes* and *D. bispora*. (**A**–**C**) Ion chromatograms depicting some of the peptides species produced by 28 days liquid cultures of the respective fungi. In case of *O. olearius* (**A**) and *D. bispora* (**B**), both backbone-N-methylated macrocyclic peptides (M + H^+^) and additionally oxidized macrocyclic peptides (M + nO + H)^+^ thereof were observed in the culture supernatant. In case of the *L. edodes* liquid culture (**C**)*,* oxidized cyclic peptides were detected in the fungal mycelium. (M + 2HO + H)^+^ represents the linear form of the macrocyclic peptide. (**D**) Chemical structures of omphalotin A, lentinulin A and dendrothelin A. Residues different from omphalotin A are underlined. Backbone N-methylation is indicated by green circles.

Since macrocyclic omphalotin A is known to undergo additional modifications including oxidation and acetylation in long-term cultures of *O. olearius*, resulting in a variety of reported omphalotin species (B-I)^18,21^, the liquid cultures were grown for 28 days and analysed for analogously modified species of lentinulin A and dendrothelin A. We detected all three macrocyclic peptides both in either the non-oxidized (omphalotin and dendrothelin) or the oxidized form (all three peptides) (Fig. 4, panels A–C). In case of omphalotins and dendrothelins, acetylated species were also detected. For *O. olearius* and *D. bispora* mycelia cultivated on solid medium, we detected omphalotin A and dendrothelin A, respectively, while for *L. edodes,* only fully methylated core peptide was observed under these conditions. The different mushroom species vary in the ratios of the different peptide species produced (Supplementary Fig. S6, panels A–C).

The presence of linear peptide species suggests that OphP (and its homologs LedP and DbiP) works the same way as GmPOPB, i.e. in a two-step fashion with an initial cleavage at the N-terminus, followed by a second cleavage at the C-terminus of the core peptide^25^. According to this mechanism, the free N-terminus of the core peptide, generated by the first cleavage, attacks the covalent intermediate of the second cleavage, which results in macrocyclization of the core peptide. The linear peptide variants are most likely attributed to the nucleophilic attack of a water molecule on the peptide's C-terminal amide linkage. In all investigated mushroom species, the full-length and the shorter linear peptides are generated by cleavage at positions G411 and G408, respectively. Interestingly, the following residues are not methylated, allowing cleavage at this position and explaining how the system directs the proteolytic release of the core peptide. Short macrocyclic peptides were not detected, potentially due to increased steric hindrance disfavoring macrocylization by the prolyloligopeptidase. In light of the byproducts detected both in the heterologous expression system and the mushrooms themselves, their physiological significance as bioactive compounds remains to be investigated.

The activity of OphP towards the 46 kDa protein OphMA is remarkable as prolyloligopeptidases commonly recognize and process short peptides of less than 35 amino acids^31,32^. In addition to the size, also the backbone N-methylation of the OphP substrate is unprecedented. In this regard, the selectivity of OphP towards fully methylated core peptide has not been explored so far and, thus, the detection of short linear peptides is highly interesting. While the three-dimensional structure of OphMA^22^ already shed some light on the mode of action and promiscuity of this enzyme, structural and mechanistic insight into the action of OphP are essential to complete the full picture. The molecular understanding of the activities of OphMA and OphP is important to exploit these enzymes for biotechnological applications. Such applications are, besides the production and characterization of novel borosin natural products, the production of libraries of new-to-nature multiply backbone N-methylated peptide macrocycles as such peptides likely possess favorable pharmacological properties and, thus, represent promising leads for novel peptide therapeutics. A biotechnological production platform, being cost-effective and environmentally friendly, would be a highly competitive alternative to the laborious chemical synthesis of such peptides^13,33^.

## Conclusions

We successfully produced omphalotin A and the two homologous peptide natural products lentinulin A and dendrothelin A in the yeast *P. pastoris* by coexpressing the prolyloligopeptidase OphP and the precursor (methyltransferase) protein OphMA or hybrids thereof. The novel dodecapeptides are highly similar in sequence, macrocyclic and ninefold backbone N-methylated. Furthermore, we also detected methylated linear nona- and dodeca-peptides, probably arising from cleavage of the methylated core-CRS precursor peptides after position nine rather than twelve and/or failure of macrocyclization of the methylated core peptide by the prolyloligopeptidase. Importantly, we show that most of these and further modified (oxidized) peptide species are also produced by the original hosts, the mushrooms *O. olearius*, *L. edodes* and *D. bispora*. Similarly to omphalotin A, the novel peptides display toxicity against the root-knot nematode *M. incognita*. The differences in bioactivity between the three peptides allow conclusions about the functional importance of specific residues*.* In summary, our synthetic biology platform for the biotechnological production of these multiply backbone N-methylated peptide macrocycles will help to elucidate the mode of action of these natural nematicides and pave the way for the production of borosin peptide natural products and their potential application as novel biopharmaceuticals and biopesticides.

## Materials and methods

### Cells, antibodies and reagents

*Pichia pastoris* strain GS115 was ordered from Invitrogen. *Omphalotus olearius* (strain DSM3398) was ordered from the German Collection of Microorganisms and Cell Cultures GmbH (DSMZ; Leibniz Institute, Germany), *Dendrothele bispora* (strain CBS 962.96) from the Westerdijk Fungal Biodiversity Institute strain collection (The Netherlands), and *Lentinula edodes* (strain 4312 from Sylvan, China) was a gift from a local mushroom farm (Fine Funghi AG, Gossau ZH, Switzerland). Zeocin powder was ordered from InvivoGen (InvivoGen Europe, Toulouse, France), geneticin G418 sulfate from GIBCO ((ThermoFisher Scientific, USA), and ampicillin sodium salt BioChemica from AppliChem GmbH (Darmstadt, Germany). Anti-strep antibody was supplied by Bio-Rad (USA), HRP-conjugated goat anti-mouse IgG by Santa Cruz Biotechnology, and anti-His4 by Qiagen. Ethyl acetate, methanol, and n-hexane were ordered from Sigma Aldrich (Buchs, Switzerland). Glass beads were obtained from Biospec Products (Bartlesville, USA). The expression vectors pPIC3.5 K, Phusion high-fidelity DNA polymerase and DreamTaq PCR Master Mix were provided by ThermoFisher Scientific (USA).

### Plasmid construction

The *ophMA* cDNA from *O. olearius* was obtained from a plasmid PMA1004 (pET24-His8-OphMA_cDNA) described earlier^21^ for expressing OphMA in *E.coli*. The cDNA was PCR-amplified using primers OphMA_Fw and OphMA_Rv, and ligated into *EcoR*I and *Not*I cloning sites of pPICZA vector to generate plasmid pPICZA-HisOphMA. The ledMA cDNA was amplified from *L. edodes* cDNA^23^, using primers LedMA-cDNA_Fw1, LedMA-cDNA_Fw2, and LedMA-cDNA_Rv-*Not*I using DreamTaq Green PCR Master Mix (2X). The resulting fragment was ligated into *NotI* and *PmII* sites of pPICZA to yield plasmid pPICZA-HisLedMA. To construct plasmids encoding OphMA hybrids with the C-terminus exchanged by that of the homologous proteins from *D. bispora* (DbiMA1) and *L. edodes* (LedMA), the *ophMA* gene was amplified using primer OphMA_Fw together with primers OphMA-DbiCterm-Rv, OphMA-LedCterm-Rv, OphMA-DbiCore_Rv and OphMA-LedCore_Rv. The four PCR fragments obtained were ligated into *EcoRI* and *NotI* sites of pPICZA vector to produce pPICZA-HisOphMA_DbiCterm, pPICZA-HisOphMA_LedCterm, pPICZA-HisOphMA_LedCORE, pPICZA-HisOphMA_DbiCORE, respectively. The plasmids were transformed into *E. coli* DH5α, and the sequences were confirmed by Sanger sequencing (Microsynth AG, Switzerland).

For the heterologous expression of prolyloligopeptidase from *L. edodes (*LedP) with SUMOSTAR fused to its N-terminus, the coding sequence of *L. edodes* Le(Bin) 0899 ss11 v1.0 was codon-optimized for the expression in *Pichia* and synthesized by BaseClear (Netherlands). StrepII tag was included at the N-terminus by a two-round PCR, using primers StrepSUMOstar-Fw1, StrepSUMOstar-Fw2 and Led-RV. First StrepSUMOstar-Fw1 and LedP-RV were used for the first round PCR, and then StrepSUMOstar-Fw2 and Led-RV for the second PCR round. The PCR product was digested with *NotI* and *BamHI,* then ligated into pPIC3.5 K vector to generate pPIC35K‐strepSUMOstar‐LedP. The latter plasmid was used to make pPIC35K‐sSUMOstar‐OoOphP by exchanging *LedP* gene fragment with *OphP* gene fragment, previously obtained from *O. olearius* cDNA^21^. The plasmids were transformed into *E. coli* DH5α, and confirmed by Sanger sequencing. The protein sequences and the oligonucleotide sequences are listed in Supplementary Tables S1 and S2, respectively.

### *P. pastoris* transformation

*Pichia pastori*s GS115 cells were transformed by electroporation using plasmid linearized by either *Pme*I or *Bsp*EI for the integration in the *AOX1* and *HIS4* locus, respectively, according to Invitrogen *Pichia* Expression Kit K1710-01 and K1740-01. Transformants were selected on YPD complete medium containing 100 μl/ml zeocin (integration of pPICZA at *AOX1*-locus) or SD-HIS minimal medium (integration of pPIC3.5 K at *HIS4*-locus). Latter transformants were subsequently screened for high copy number integrants on YPD complete medium containing 200 μ/ml geneticin. To produce omphalotin A, *P. pastoris* GS115 strain already expressing OphMA (GS115-OphMA) was transformed with pPIC3.5 K carrying *OphP* gene, while for other peptides, Pichia strain stably expressing OphP or LedP were transformed with pPICZA vector harboring *ophMA* hybrid genes. Positive clones were enriched by selection on SD-HIS medium containing 200 µg/ml zeocin followed by selection on SD-HIS with 200 μg/ml geneticin. In all cases, integration and expression of the gene of interest was confirmed by PCR and immunoblot, respectively (Supplementary Fig. S2).

### Protein production in *P. pastoris*

To produce the recombinant proteins in *P. pastoris*, cells were handled according to to Invitrogen *Pichia* Expression Kit K1710-01 and K1740-01. Briefly, cells were first cultured in buffered glycerol-containing complex medium (BMGY) until the OD_600_ reached between 2 and 6. Cells were washed and cultured in methanol-containing complex medium (BMMY) for three days. To maintain the induction, 0.5% methanol (v/v) was supplied each 24 h. Cells were separated from the medium by centrifugation and resuspended in the lysis buffer composed of 2 × PBS supplemented with 0.2 mg/l PMSF and protein inhibitor cocktail (PIC) tablets, EDTA free, Roche. The cell suspension was saturated with 0.5 mm diameter glass beads, and the cells were lysed using a planetary mill (Pulverisette 7, Fritsch, Germany) at level 3, 400 rpm and 3 repetitions. After centrifugation at 8000 g for 30 min, the supernatant was collected and further used for protein purification by Ni–NTA beads or peptide extraction (see below).

### Tryptic digestion

After protein extraction and size exclusion chromatography, the purified precursor proteins were concentrated up to 4 μg/μl. 25 μL of the protein (100 μg) place in a in a V-shaped glass viol and add 1.5 μl trypsin (80:1), and incubated overnight at 37° C for 4-5 h or overnight, 650 rpm. 3.0 μL of the sample were used for LC–MS analysis.

### Extraction of peptides from *P. pastoris*

The cleared cell lysate and the culture supernatant were vigorously mixed with the same volume of ethyl acetate (1:1, v/v) in a separatory funnel. The organic layer was collected and evaporated using a rotary evaporator. The resulting pellets were dissolved into 100% methanol. To purify peptides from *Pichia* cell extracts, reversed phase HPLC was performed using mobile phase made of solvent A (water) and solvent B (acetonitrile) over a C18 column (Phenomenex Luna 5μ C18, 10 × 250 mm) with a flow rate of 12 ml/min. The solvent gradient started from equilibrating the column at 40% B for 3 min, then linear gradients from 40 to 100% B for 37 min, followed by washing the column at 100%B for 10 min. The peptide peaks were observed at λ210 nm after 28.5 min, 32.5 min and 35 min for dendrothelin A, lentinulin A and omphalotin A, respectively. The respective fractions were collected, evaporated, and weighed.

### Cultivation of fungal mycelium and extraction of peptides thereof

Mycelia of *Omphalotus olearius, Dendrothele bispora*, and *Lentinula edodes* freshly grown on YMG (0.4% (w/v) yeast extract, 2% (w/v) malt extract, 0.4% (w/v) glucose, 1.5% (w/v) agar) plates were used to inoculate liquid and solid cultures. For culturing on solid medium, eight plates with cellophane were used for each fungal species. Small blocks of mycelia were placed on each plate, and then plates were incubated for 10 to 12 days at 23 °C in aerated dark humid chambers. Mycelia were scraped from plates and then distributed in screw cap tubes, and lysed with 0.5 mm glass beads in 2 × PBS containing Roche DNAse using a Thermo FastPrep FP120 cell disruptor. After centrifugation at 20,000 xg, the cleared cell lysate was adjusted to 50 ml with 2 × PBS, and peptides were extracted using ethyl acetate (1:1). For liquid cultures, 500 ml YMG was used for *O. olearius* and YMMG (double amount of malt extract) for *L. edodes* and *D. bispora.* Cultures were cultivated for 28 days at 23 °C. While cultures of *O. olearius* and *D. bispora* were shaken at 180 rpm, *L. edodes* was grown in standing cultures. The culture media were cleared by centrifugation and then used for peptide extraction. In all cases, after evaporating the solvents, the pellets were dissolved in methanol.

### Peptide detection by LC–MS/MS

LC–MS/MS spectra were acquired on a Thermo Scientific Q Exactive classic using heated electrospray ionization in positive ion mode coupled to a Dionex Ultimate 3000 UHPLC using a Phenomenex Kinetex ®µm XB-C18 100 Å (150 × 2.1 mm) column heated at 50 °C. Mobile phase was made of solvent A (water with 0.1% formic acid: v/v) and solvent B (acetonitrile with 0.1% formic acid: v/v). We used two different LC–MS/MS methods. Method 1 was performed to detect methylated core peptides released by prolyloligopeptidase. The HPLC gradient for method 1 was set with a constant flow rate of 0.5 ml/min. the column was equilibrated 30% B for 5 min, followed by a linear gradient to 95% B for 15 min, then flushing the column with 100% B for 7 min, and finally re-equilibrating the column at 30% B for 5 min. The following conditions were used for heated electrospray ionization (HESI): capillary temperature, 269 °C; sheath gas, 53 arbitrary units; auxiliary gas, 14 units; probe heater temperature, 438 °C; S-Lens level, 50%; and spray voltage, 3.5 kV. Full MS was done at a resolution of 70,000 (AGC target 1e6, maximum IT 100 ms, scan range 300–2000 m/z). Data dependent MS/MS was performed at a resolution of 17,500 (AGC target at 1e5, maximum IT 120 ms, isolation windows in the range of 3.0 m/z). The normalized collision energy (NCE) was set at 30. Method 2 was used for analysis of C-terminal tryptic peptides from OphMA and hybrids. The flowrate was set at 0.8 ml/min with different gradients of solvent B: 0% to 5% for 2 min, 5% to 85% for 13 min, and from 85 to 98% for 2 min, column flushing at 98% for 4 min, and reequilibration at 5% for 2.5 min. HESI parameters were set as follows: auxiliary gas, 18 units; probe heater temperature, 475 °C; S-Lens level, 50%; and spray voltage, 3.5 kV. A capillary temperature of 288 °C; sheath gas of 60 units. Full MS was done at a resolution of 35,000 (AGC target 1e6, maximum IT 750 ms, scan range 340–2300 m/z). Data dependent MS/MS was performed at a resolution of 17,500 (AGC target at 1e6, maximum IT 500 ms, isolation windows in the range of 2.0 m/z) using a stepped NCE of 14, 16 and 20. Data was processed using Thermo Fisher Xcalibur software.

### *M. incognita* cultivation and in vitro nematotoxicity assay

*Meloidogyne incognita* was reared in the greenhouse on tomato plants (*Solanum lycopersicum* cv. Oskar) at 24 ± 2 °C and 60% humidity with a day/night cycle of 15:9 h. Second-stage juveniles (J2) were extracted from galled up tomato roots, placed under the mist chamber^28^. Freshly hatched J2 were collected daily and stored in the fridge (6 °C) for up to five days before quantifying the nematodes under a 40 × magnification light microscope for the preparation of the nematode suspension. The inhibitory effect of omphalotin A, lentinulin A and dendrothelin A on *M. incognita* J2 after 1, 3, 7 and 14 days of exposure was tested in a 24-well microtiter plate. The compounds were solubilized in methanol and adjusted to a final concentration of 0.1 µM, 0.75 µM, 1.5 µM and 3 µM (n = 3; final) to each well. Methanol was then evaporated, and each well was filled with 2 mL of a nematode suspension containing 200 J2 M*. incognita.* The first 100 J2 per well were scored for normal motility, affected motility, and immotile (elongated). H_2_O was used as negative control and the nematicide Fluopyram (Velum Prime^R^) was used as positive control.

## Supplementary Information

Supplementary Information 1.

Supplementary Information 1.
