# Author Correction: Identifcation, heterologous production and bioactivity of lentinulin A and dendrothelin A, two natural variants of backbone N-methylated peptide macrocycle omphalotin A

**DOI:** 10.1038/s41598-021-91767-2

**Published:** 2021-06-10

**Authors:** Emmanuel Matabaro, Hannelore Kaspar, Paul Dahlin, Daniel L. V. Bader, Claudia E. Murar, Florian Staubli, Christopher M. Field, Jeffrey W. Bode, Markus Künzler

**Affiliations:** 1 Department of Biology, Institute of Microbiology, ETH Zürich, Room HCI F409, Vladimir-Prelog-Weg 4, CH-8093 Zürich, Switzerland; 2 Agroscope, Phytopathology and Zoology in Fruit and Vegetable Production, Müller-Thurgau-Strasse 29, CH-8820 Wädenswil, Switzerland; 3 Department of Chemistry and Applied Biosciences, Laboratorium Für Organische Chemie, ETH-Zürich, Vladimir-Prelog-Weg 3, CH-8093 Zürich, Switzerland; 4 Institute of Transformative Bio-Molecules (WPI-ITbM), Nagoya University, Chikusa, Nagoya, 464-8602 Japan

Correction to: *Scientific Reports* 10.1038/s41598-021-83106-2, published online 11 February 2021

The original version of this Article contained an error in Figure 4d where the stereochemistry of the residues of the macrocyclic peptides was incorrect.

In addition, in the legend of Figure 4,

“In case of the *L. edodes* liquid culture (**C**), oxidized cyclic peptides were detected in the fungal mycelium. (M + 2HO + H)+ represents the linear form of the macrocyclic peptide.”

now reads:

“In case of the *L. edodes* liquid culture (**C**), oxidized cyclic peptides were detected in the fungal mycelium. (M + H_2_O + H)+ represents the linear form of the macrocyclic peptide.”

The original Figure 4 and accompanying legend appear below. The original Article has been corrected.

**Figure 4:**
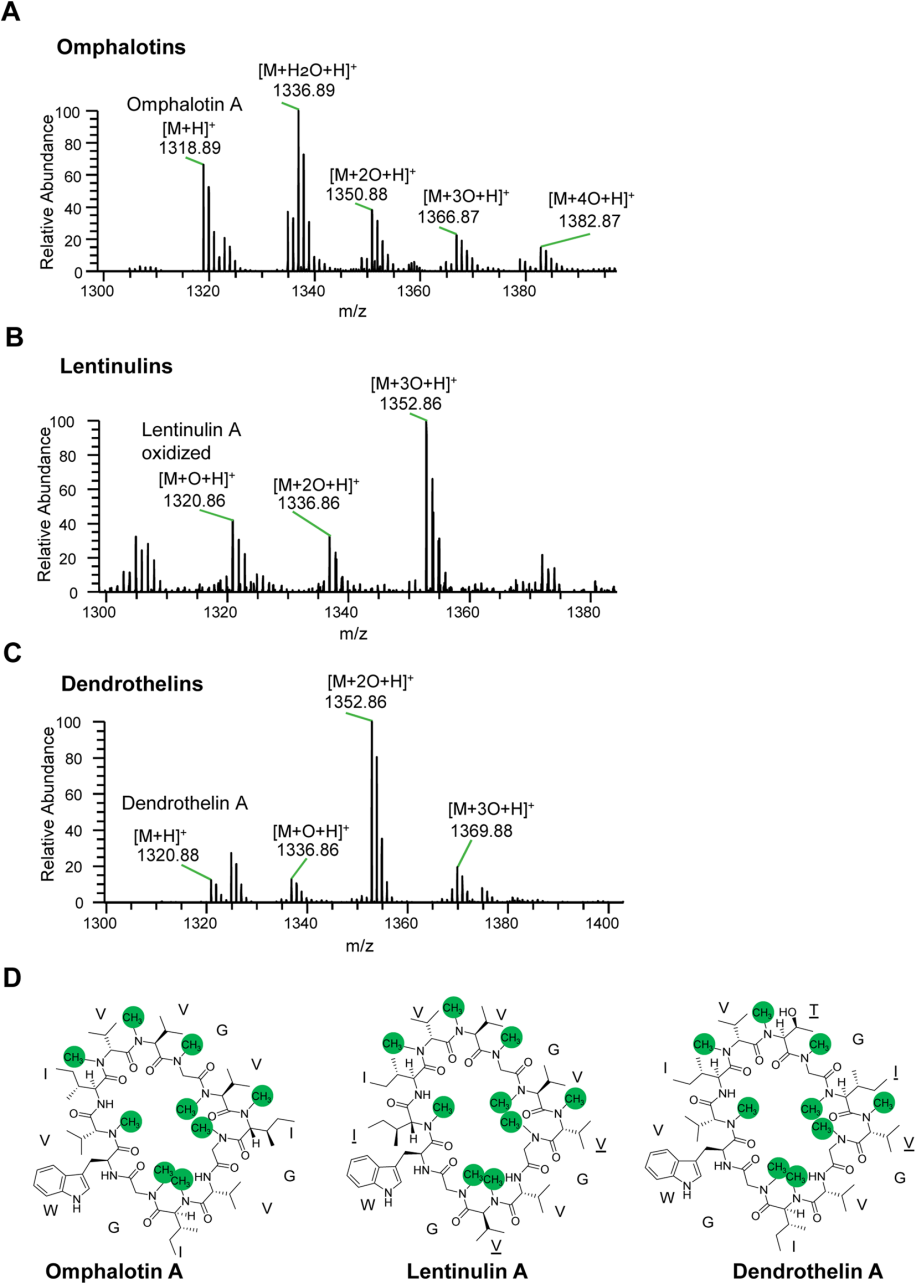
LC–MS analysis of backbone N-methylated peptides extracted from mushrooms *O. olearius*, *L. edodes* and *D. bispora*. (**A**–**C**) Ion chromatograms depicting some of the peptides species produced by 28 days liquid cultures of the respective fungi. In case of *O. olearius* (**A**) and *D. bispora* (**B**), both backbone-N-methylated macrocyclic peptides (M + H^+^) and additionally oxidized macrocyclic peptides (M + nO + H)^+^ thereof were observed in the culture supernatant. In case of the *L. edodes* liquid culture (**C**)*,* oxidized cyclic peptides were detected in the fungal mycelium. (M + 2HO + H)^+^ represents the linear form of the macrocyclic peptide. (**D**) Chemical structures of omphalotin A, lentinulin A and dendrothelin A. Residues different from omphalotin A are underlined. Backbone N-methylation is indicated by green circles.

