# Author Correction: Identification, heterologous production and bioactivity of lentinulin A and dendrothelin A, two natural variants of backbone N-methylated peptide macrocycle omphalotin A

**DOI:** 10.1038/s41598-022-21642-1

**Published:** 2022-10-11

**Authors:** Emmanuel Matabaro, Hannelore Kaspar, Paul Dahlin, Daniel L. V. Bader, Claudia E. Murar, Florian Staubli, Christopher M. Field, Jeffrey W. Bode, Markus Künzler

**Affiliations:** 1 Department of Biology, Institute of Microbiology, ETH Zürich, Room HCI F409, Vladimir‑Prelog‑Weg 4, CH‑8093 Zürich, Switzerland; 2 Agroscope, Phytopathology and Zoology in Fruit and Vegetable Production, Müller‑Thurgau‑Strasse 29, CH‑8820 Wädenswil, Switzerland; 3 Department of Chemistry and Applied Biosciences, Laboratorium Für Organische Chemie, ETH-Zürich, Vladimir‑Prelog‑Weg 3, CH‑8093 Zürich, Switzerland; 4 Institute of Transformative Bio‑Molecules (WPI‑ITbM), Nagoya University, Chikusa, Nagoya Japan

Correction to: *Scientific Reports* 10.1038/s41598-021-83106-2, published online 11 February 2021

The original version of this Article contained errors in Figures 1 and 2, where the methylation patterns of the peptide sequences were incorrect in Figure 1 panel (c) and Figure 2 panel (a). The original Figures 1 and 2 and accompanying legends appear below.

**Figure 1:**
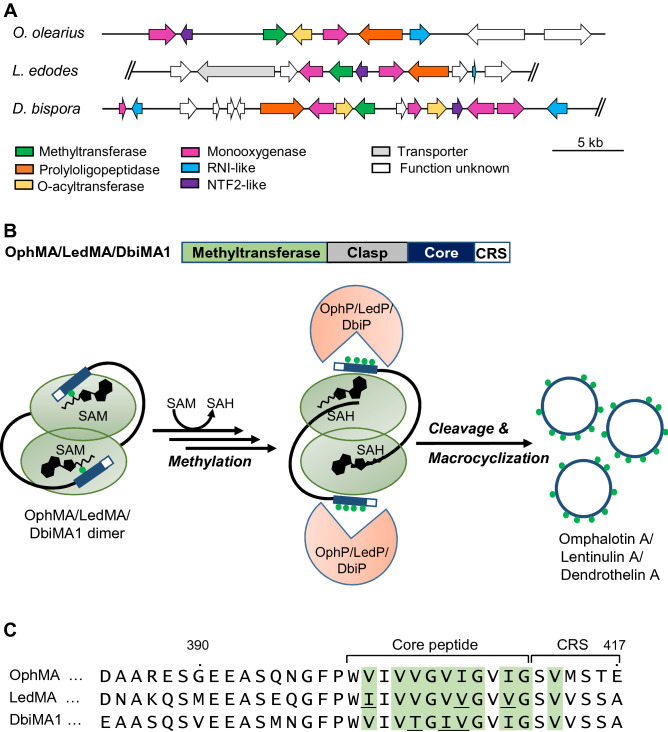
Structure and function of omphalotin biosynthetic gene cluster and homologous clusters in the basidiomycetes *O. olearius*, *D. bispora* and *L. edodes.* (**A**) Schematic representation of the borosin biosynthetic gene clusters in the basidiomycetes *O. olearius* (VT 653.13, scaffold*_*169), *L. edodes* (Le(Bin) 0899 ss11, scaffold_10) and *D. bispora* (CBS 962.96, scaffold_621). All three clusters code for a precursor (methyltransferase) protein, referred to as OphMA, LedMA and DbiMA1 respectively, and a prolyloligopeptidase, referred to as OphP, LedP and DbiP, respectively. The DNA scaffolds and filtered gene models were taken from https://mycocosm.jgi.doe.gov/. Double slashes (//) indicate that the sequence of the DNA scaffold continues beyond this position. (**B**) Biosynthesis scheme of omphalotin A and closely related peptides, lentinulin A and dendrothelin A as a combined action of the precursor (methyltransferase) protein and the prolyloligopeptidase. (**C**) Alignment of the C-termini of the precursor (methyltransferase) proteins from *O. olearius* (OphMA), *L. edodes* (LedMA) and *D. bispora* (DbiMA1). Residues differing from the omphalotin core peptide are shown in red. The indicated methylation patterns (green fill) were previously determined by heterologous expression of the respective cDNAs in *E. coli*^21,23,25^. *CRS* C-terminal recognition sequence.

**Figure 2:**
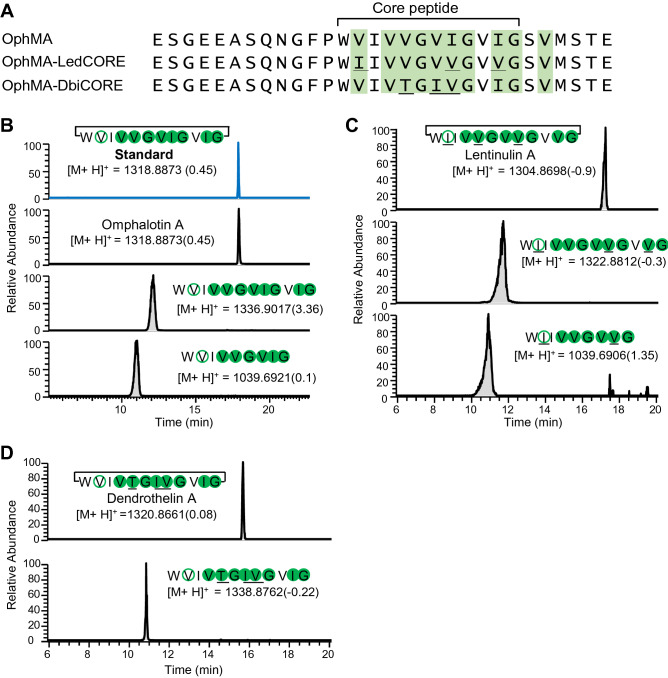
Production of backbone N-methylated peptide macrocycles in *P. pastoris*. (**A**) Methylation pattern of OphMA, OphMA_LedCORE and OphMA_DbiCORE. Methylated residues were determined by LC–MS/MS analysis of the C-terminal tryptic fragments and are shaded in green (Supplementary Fig. S2A–C). (**B**) Production of omphalotin A. The extracted compound was detected using LC–MS and compared to the chemically synthesized omphalotin A standard (216 ng/ml). In addition to omphalotin A, fully methylated, linear (core) peptides and short linear peptides lacking the C-terminal three residues (VIG) were detected. (**C**) Production of lentinulin A and (**D**) dendrothelin A. The detected peptides were confirmed by LC–MS/MS. The confirmed positions of methylation are depicted using filled circles, while methylated residues inferred from MS/MS are shown in open circles. Residues different from omphalotin A are underlined. The mass difference (represented in ppm) between observed values and theoretical mass is indicated in brackets for each compound.

The original Article has been corrected.

