# Supplementary material for: Identification, heterologous production and bioactivity of lentinulin A and dendrothelin A, two natural variants of backbone N-methylated peptide macrocycle omphalotin A
Source: Sci Rep. 2021 Feb 11;11:3541. doi: 10.1038/s41598-021-83106-2 (PMC7878506; doi:10.1038/s41598-021-83106-2)
Supplement: Supplementary file 1 — Supplementary Information 1. [file 41598_2021_83106_MOESM1_ESM.pdf]

SUPPLEMENTARY INFORMATION

Identification, heterologous production and bioactivity of lentinulin A and dendrothelin A, two natural variants of backbone N-methylated peptide macrocycle omphalotin A

Emmanuel Matabaro[1], Hannelore Kaspar[1], Paul Dahlin[2], Daniel L. V. Bader[3], Claudia E. Murar[3], Florian Staubli[1], Christopher M. Field[1], Jeffrey W. Bode[3,4] & Markus Künzler[1,*]

[1]ETH Zürich, Department of Biology, Institute of Microbiology, Vladimir-Prelog-Weg 4, CH-8093 Zürich, Switzerland

[2]Agroscope, Phytopathology and Zoology in Fruit and Vegetable Production, Müller-Thurgau-Strasse 29, CH-8820 Wädenswil, Switzerland

[3]ETH-Zürich, Department of Chemistry and Applied Biosciences, Laboratorium für Organische Chemie, Vladimir-Prelog-Weg 3, CH-8093 Zürich, Switzerland

[4]Institute of Transformative Bio-Molecules (WPI-ITbM), Nagoya University, Chikusa, Nagoya 464-8602, Japan

[*]Correspondence and requests for materials should be addressed to M.K.

Room HCI F409

Phone +41 44 632 49 25

E-mail: mkuenzle@ethz.ch

Contents

Supplementary Materials and methods: Chemical synthesis of omphalotin A 3
Supplementary Figure S1. Production and methylation pattern of wild type OphMA and hybrids thereof in *P. pastoris* 7
Supplementary Figure S2. Genomic integration and coexpression of *ophMA* and *ophP* in *P. pastoris* 8
Supplementary Figure S3. Production of omphalotin A, lentinulin A and dendrothelin A in *P. pastoris* 11
Supplementary Figure S4. MS/MS spectral annotation of peptides produced in *P. pastoris*. 15
Supplementary Figure S5. Cross-reactivity of OphP homologues from different fungi in *P. pastoris* 17
Supplementary Figure S6. Relative abundance of peptide species extracted from cultures of the original host 18
Supplementary Figure S7. HPLC chromatograms of the purified recombinant peptides 19
Supplementary Figure S8. Estimation of LC50 for different treatments at different time points. 20
Supplementary Table S1. List of protein sequences 21
Supplementary Table S2. List of oligonucleotides 22

Supplementary Materials and methods: Chemical synthesis of omphalotin A

***General information*.** HPLC grade acetonitrile from Sigma-Aldrich (Buchs, Switzerland) was used for analytical and preparative purification. DMF and DIPEA from Sigma-Aldrich (Buchs, Switzerland) for solid phase peptide synthesis were used without additional purification. Additional commercially available reagents and solvents were purchased from Sigma-Adrich (Buchs, Switzerland), except stated otherwise. Solvents for flash chromatography (EtOAc, MeOH) were of technical grade and used without further purification. Coupling reagents, such as HATU and all Fmoc-amino acids were purchased from Peptides International (Louisville, KY, USA) and Chemimpex (Wood Dale, IL, USA). 2-Chlorotrityl chloride resin (100-200 mesh) was purchased from Novabiochem (Burlington, MA, USA).

***Reactions and purification*.** Peptides were purified on Jasco reverse phase high performance liquid chromatography (RP-HPLC) preparative apparatus equipped with a dual PU 2087 plus pumps, in-line degasser and the spectra were recorded simultaneously at three different wavelengths: 220 nm, 254 nm and 301 nm with a UV-2077 plus detector. The mobile phase was composed of high-grade Millipore H_2O and acetonitrile containing 0.1% (v/v) TFA. At a flow rate of 10 mL/min using a Phenomenex C4 (10 μm, 100 Å, 250 x 21 mm) column. The column was heated to 60°C and pre-equilibrated at 40% acetonitrile for approximately 10 min before injecting the sample. After 5 min at 40% acetonitrile a linear gradient was run for 40 min until a final 95% acetonitrile gradient was reached. Finally, the columns were flushed for 7 min at 95% acetonitrile. The cyclization reaction was monitored by thin layer chromatography using pre-coated glass plates Merck (Burlington, MA, USA) and visualized by ninhydrin staining.

The synthesis of the hydrophobic cyclic peptide was performed by established Fmoc manual solid phase peptide synthesis, followed by standard resin cleavage, cyclization reaction and several purification steps[1]. To load the resin, 2-chlorotrityl resin (1 g, 1.46 mmol/g, 1.46 mmol) was supplied with 10 mL of CH_2Cl_2 and vigorously shaken for 30 min at room temperature. The swollen resin was further rinsed with CH_2Cl_2 (3 x 10 mL) and supplied with a solution of DIPEA (278.7 μL, 1.6 mmol, 1.0 eq.) activated Fmoc-Sar-OH (124.5 mg, 0.4 mmol, 0.274 eq.) in 10 mL of CH_2Cl_2. The resin was shaken for 2 h at room temperature, before being thoroughly washed with CH_2Cl_2 (5 x 10 mL), DMF (5 x 10 mL), followed by CH_2Cl_2 (5 x 10 mL). The resin was further flushed with 20 mL of diethyl ether and dried under nitrogen flux. After determining the resin loading (0.3112 mmol/g), the resin was swollen in 10 mL CH_2Cl_2 for 30 min, flushed and supplied with 10 mL capping solution CH_2Cl_2/MeOH/DIPEA (17:2:1) for 5 min at room temperature. This process was repeated once. The resin was washed thoroughly with CH_2Cl_2 (5 x 10 mL), DMF (5 x 10 mL), followed by CH_2Cl_2 (5 x 10 mL) and dried under nitrogen flux. Loaded resin was stored in the fridge overnight.

***Loading efficiency calculation*.** After the addition of the first amino acid, approximately 12.3 mg of dried resin were collected and subjected to 2 mL 2% DBU in DMF, shaken for 15 min at room temperature and filtered through cotton. 2 x 3 mL solutions were prepared containing 2952 μL of acetonitrile; 48 μL of 2% DBU (background) or 48 μL cleavage solution (sample) and used for absorbance measurements. Resin loading of 0.31 mmol/g (Abs_{305nm} = 0.2334) was calculated using the following: Loading (mmol/g) = (Abs_{305nm} x 16.4)/mg of resin[2].

Subsequent amino acid couplings. The resin was swollen in DMF for 30 min at room temperature, before being supplied with 8 mL of 20% piperidine solution and shaken for 8 min. Fmoc deprotection was repeated once. Simultaneously, Fmoc amino acid (5 equiv.) and HATU (4.95 equiv.) were dissolved in 8 mL of DMF and activated with DIPEA (10 equiv.) for 3 min. The resin was vigorously washed with DMF and DCM and supplied with the activated

amino acid solution. The coupling was carried out at room temperature for 3 hours and washed thoroughly. 10 mL of DMF, acetic anhydride (5 eq.) and NMM (5 eq.) were added to the resin. Capping procedure was carried out for 15 min and resin further washed with DMF (5 x 10 mL), CH_2Cl_2 (5 x 10 mL), followed by DMF (5 x 10 mL). The resin was dried under reduced pressure for 30 min and stored in the fridge.

Stepwise analysis of peptide couplings. Peptide coupling efficiencies were continuously monitored, by micro cleavage after each coupling step. Approximately 15 mg of dried resin were supplied with 1 mL of HFIP/ CH_2Cl_2 (1:5) and shaken for 30 min at room temperature. The mixture was filtered through cotton filters and solvents evaporated under reduced pressure. Crude peptides were dissolved in 500 μL acetonitrile and analyzed by HPLC and LC-MS analysis.

Cleavage of resin. 600 mg of Fmoc protected peptide resin was swollen in 10 mL of DMF for 30 min. Fmoc deprotection was performed by adding 8 mL of 20% piperidine in DMF and shaken for 5 min twice. The resin was washed thoroughly and supplied with 15 mL of HFIP/ CH_2Cl_2 (1:5) and shaken for 30 min at room temperature. The solvent was evaporated under reduced pressure.

Cyclization and purification. The following cyclization reaction was carried out as previously reported[3]. Crude linear Omphalotin A (125 mg, 91.3 μM, 1 equiv.) was dissolved in 40 mL of CH_2Cl_2. 1-Hydroxy-7-azabenotrizole (24.8 mg, 182 μmol, 2 equiv.), 1-ethyl-3-(3-dimethylaminopropyl)carbodiimde (32.3 μL, 182 μmol, 2 eq.) and DIPEA (117 μL, 730 μmol, 8 equiv.) were added to the solution and the reaction mixture was stirred for 16 hours at room temperature. After 70% of the solvent was evaporated at room temperature, the organic phase was washed twice with saturated $NaHCO_3$, once with 10% citric acid and brine. The organic fractions were collected and dried with Na_2SO_4. The solvent was evaporated under reduced pressure. The product was purified via gradient flash chromatography EtOAc/MeOH (1%-10% MeOH). The fractions were collected and the solvent was evaporated under reduced pressure to afford Omphalotin A (6.5 mg) as a white solid (calc.: $[M+H]^+$ = 1318.89 m/z; measured: $[M+H]^+$ = 1318.89 m/z). The obtained peptide was further purified by two subsequent preparative HPLC runs. 6.5 mg of cyclic compound were dissolved in 4.5 mL of DMSO and injected on the preparative HPLC (C4 column, 60°C, 40-95% gradient in 40 min). Omphalotin A was collected and lyophilized over two days.

Characterization. Peptides were analysed by JASCO analytical reverse phase high performance liquid chromatography (RP-HPLC) instrument equipped with a dual PU 2080 plus pump system and in-line degasser. Spectra were monitored simultaneously at three different wavelengths: 220 nm, 254 nm, 301 nm with a UV-2077 plus detector and mobile phase was constituted of high-grade Millipore H_2O and acetonitrile containing 0.1% (v/v) TFA. Flow rate was maintained at 1 mL/min using a Phenomenex C4 (5 μm, 250 x 4.6 mm) column. The column was pre-equilibrated at 40% acetonitrile for approximately 10 min before injecting the sample. After 5 min at 40% acetonitrile a linear gradient was run for 30 min until a final 95% acetonitrile gradient was reached. Finally, the columns were flushed for 7 min at 95% acetonitrile.

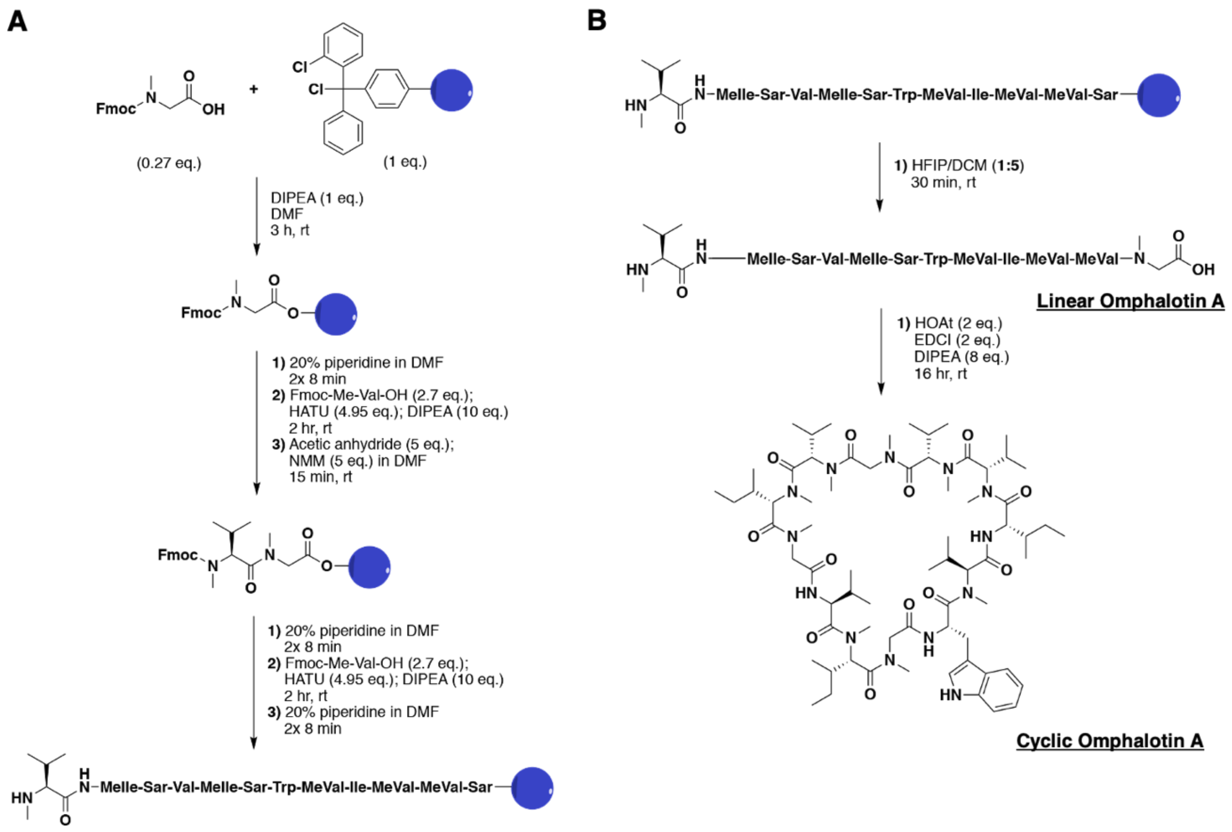

Graphical representation of the synthesis. (A) Systematic peptide coupling of Omphalotin A amino acid sequence by standard Fmoc-solid phase peptide synthesis (SPPS) on a 2-chlorotrityl chloride resin. (B) Cleavage of linear Omphalotin A of 2-CTC resin in mild acidic conditions followed by a liquid phase cyclization reaction to yield Omphalotin A.

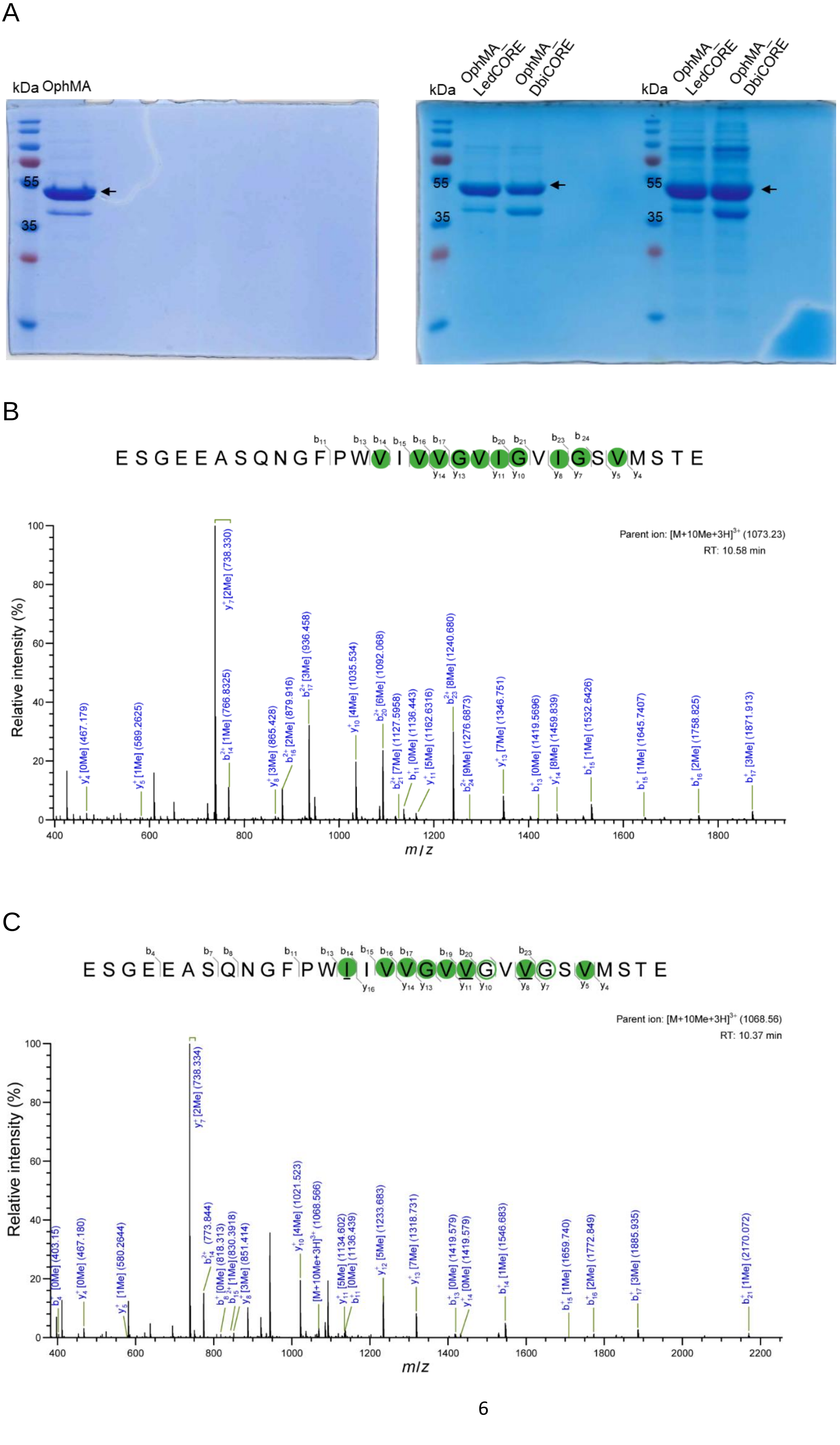
A
kDa OphMA
55
35
kDa OphMA_LedCORE OphMA_DbiCORE
55
35
kDa OphMA_LedCORE OphMA_DbiCORE
55
35
B
ESGEEASQNGFPWVIVVGVIGVIGSVMSTE
Parent ion: [M+10Me+3H]3+ (1073.23)
RT: 10.58 min
Relative intensity (%)
m/z
C
ESGEEASQNGFPWIIVVGVVGVVGSVMSTE
Parent ion: [M+10Me+3H]3+ (1068.56)
RT: 10.37 min
Relative intensity (%)
m/z

D

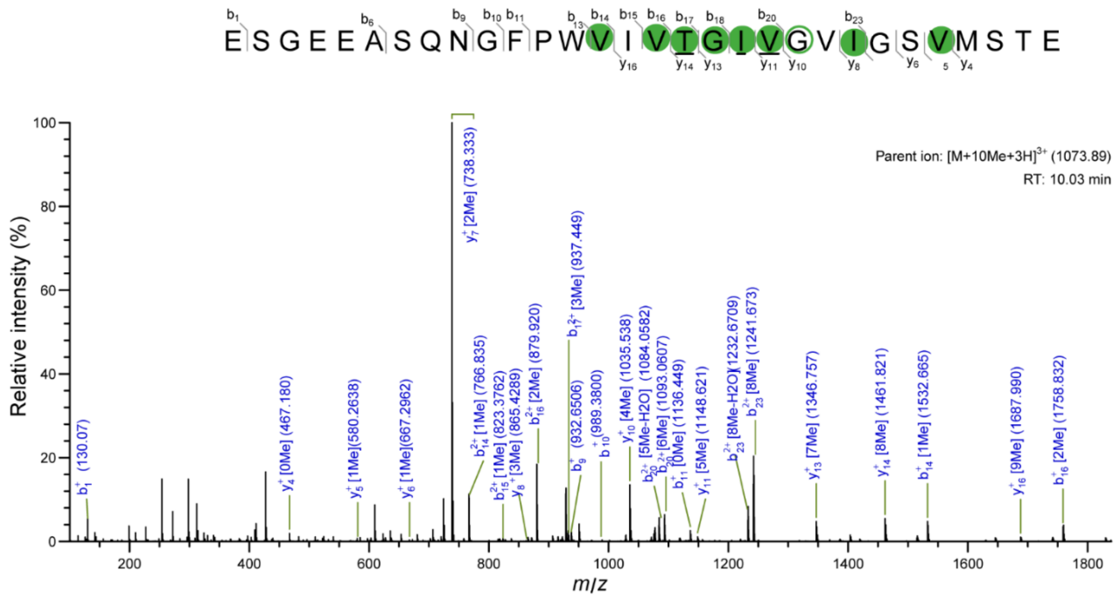

Supplementary Figure S1. Production and methylation pattern of wild type OphMA and hybrids thereof in *P. pastoris*. (A) SDS-PAGE of purified OphMA and hybrids thereof. Proteins were purified by Ni-NTA beads and subsequent size exclusion chromatography. 30 µg of purified OphMA and 15 µg (left lanes) and 30 µg (right lanes) of the purified hybrid proteins were loaded onto a 12% polyacrylamide gel and gels were stained with Coomassie brilliant blue. The molecular mass of selected marker proteins (M) is indicated in kDa. The recombinant full-length proteins are indicated by arrows. (B-D) LC-MS/MS analysis of methylation pattern of OphMA (B), OphMA_LedCORE (C) and OphMA_DbiCORE (D). Methylated residues were determined by LC-MS/MS analysis of the C-terminal tryptic fragments and are marked by green circles. Filled circles indicate positions confirmed by MS/MS, unfilled circles represent methylated residues inferred by MS and the methylation pattern of omphalotin A. Only the corresponding spectra of the tryptic fragments with 10-fold methylation, representing the most abundant species, are shown. Residues different from the omphalotin core peptide are underlined.

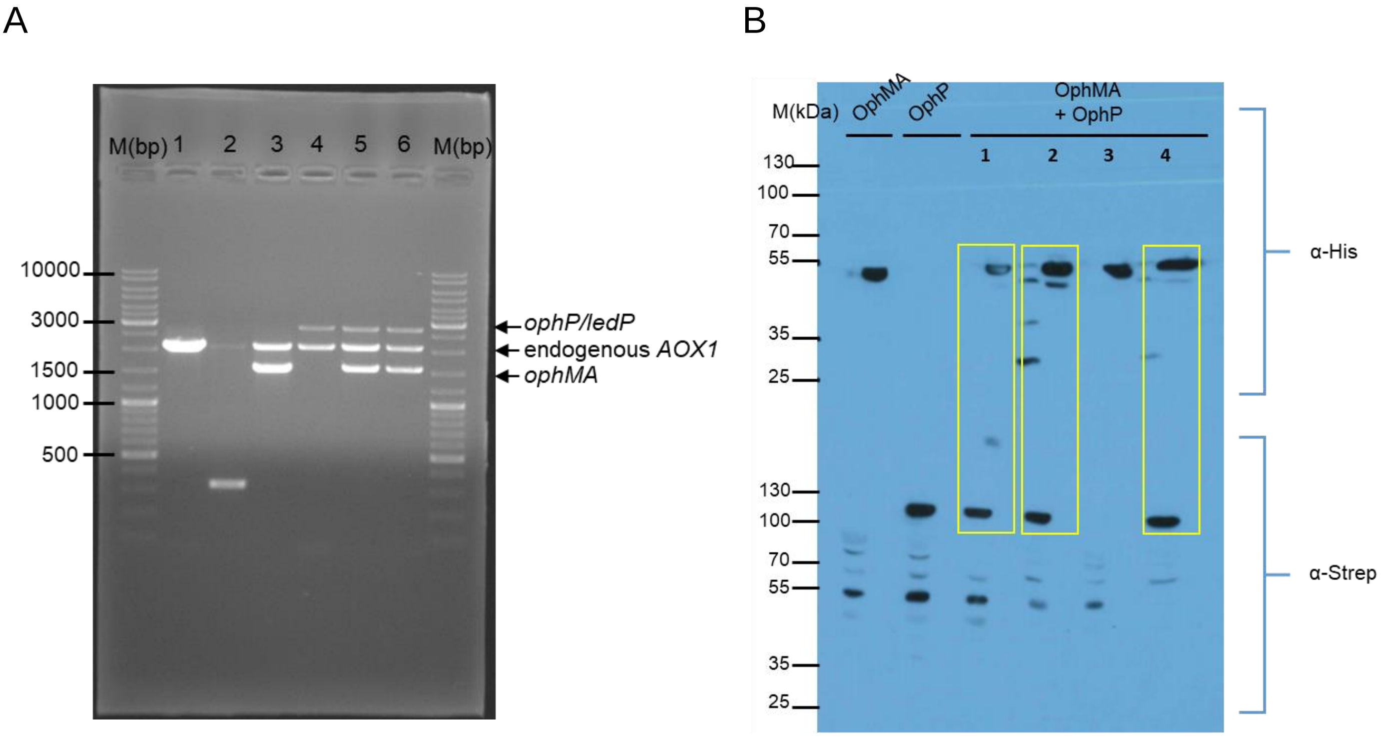

Supplementary Figure S2. Genomic integration and coexpression of *ophMA* and *ophP* in *P. pastoris*. (A) Colony PCR analysis of transformants of *P. pastoris* strain GS115 expressing *ophMA* and *ophP* alone, and coexpressing *ophMA* together with *ophP or ledP* (lane 6). The transformants were streaked on YPD plates, and incubated at 30°C for three days. One single colony for each strain was scraped off the plate using a loop and resuspended in 30 µl of 0.2% SDS in an Eppendorf tube, heated to 95°C for 10 min and centrifuged at 11000 x g for 2 min. The supernatant (0.5 µl) was used as template of a PCR reaction following the user guide of DreamTaq™ Green PCR Master Mix (2x) (ThermoFisher Scientific). Oligonucleotides 5-*AOX1* and 3-*AOX1* (see Supplementary Table 2 for sequences) were used as primers. 5 µl of the PCR reaction were run out on a 1% (w/v) agarose gel and stained with EtBr. Transformant 1, GS115; 2, GS115-pPICZA; 3, GS115-OphMA; 4, GS115-OphP; 5, GS115-OphMA-OphP; 6, GS115-OphMA-LedP. Transformants expressing the hybrid *ophMA* constructs were analyzed accordingly (data not shown). (B) Immunoblot of soluble protein extracts of respective GS115 transformants assessing the production of OphP and OphMA. Heterologously produced OphMA was N-terminally His-tagged while OphP was fused to a N-terminal StrepII-SUMOSTAR. Anti-His-tag (α-His) and anti-StrepII-tag (α-Strep) were used as primary antibodies, while HRP-conjugated goat anti-mouse IgG served as secondary antibodies. The yellow boxes highlight the concomitant production of OphMA and OphP in three different GS115-OphMA-OphP transformants (1, 2). Transformants expressing the hybrid *ophMA* and the *ledP* constructs were analyzed accordingly (data not shown).

A

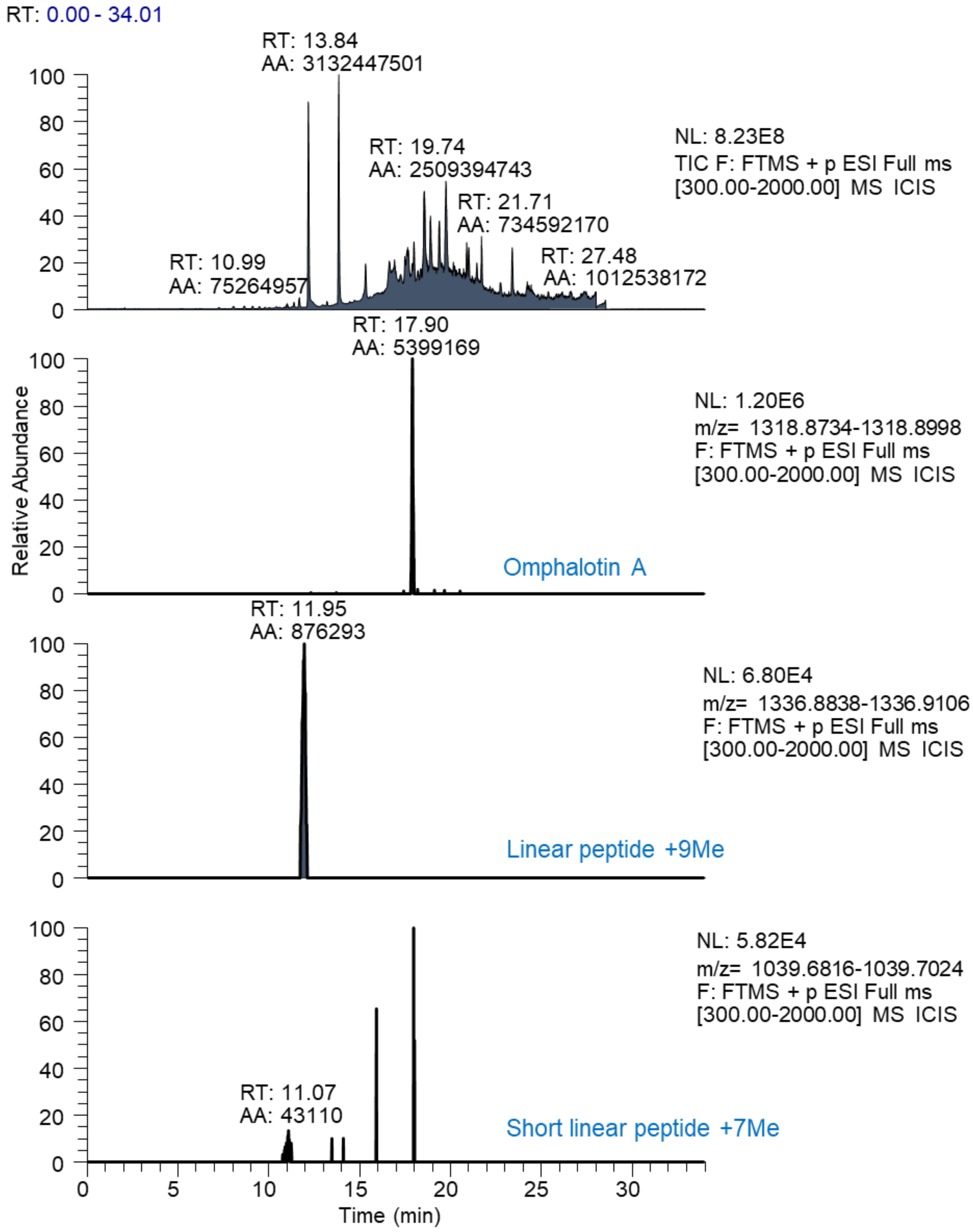
RT: 0.00 - 34.01
RT: 13.84
AA: 3132447501
RT: 19.74
AA: 2509394743
RT: 21.71
AA: 734592170
RT: 10.99
AA: 75264957
RT: 27.48
AA: 1012538172
NL: 8.23E8
TIC F: FTMS + p ESI Full ms
[300.00-2000.00] MS ICIS
RT: 17.90
AA: 5399169
NL: 1.20E6
m/z= 1318.8734-1318.8998
F: FTMS + p ESI Full ms
[300.00-2000.00] MS ICIS
Omphalotin A
RT: 11.95
AA: 876293
NL: 6.80E4
m/z= 1336.8838-1336.9106
F: FTMS + p ESI Full ms
[300.00-2000.00] MS ICIS
Linear peptide +9Me
NL: 5.82E4
m/z= 1039.6816-1039.7024
F: FTMS + p ESI Full ms
[300.00-2000.00] MS ICIS
RT: 11.07
AA: 43110
Short linear peptide +7Me
Relative Abundance
Time (min)

B

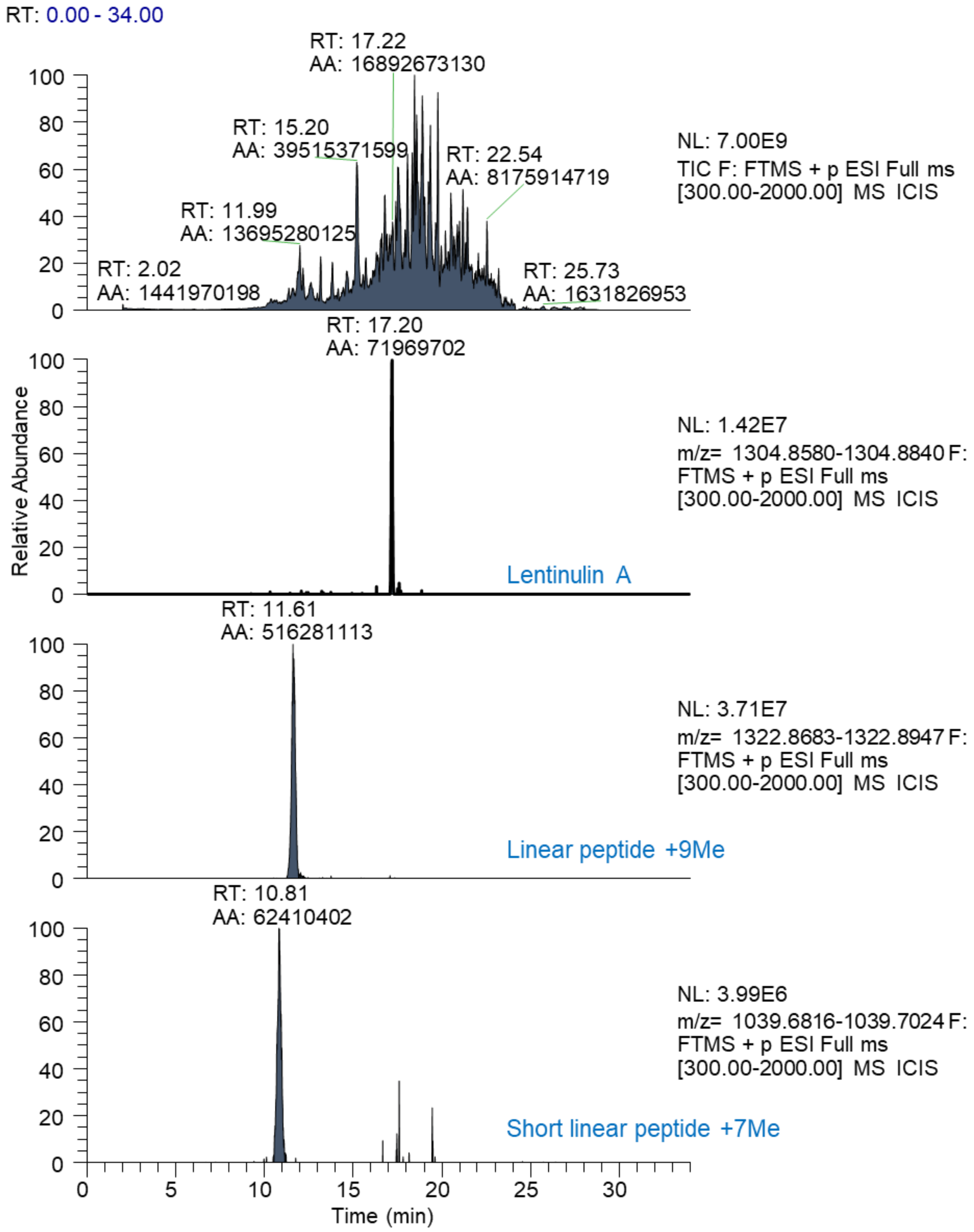

RT: 0.00 - 34.00
RT: 17.22
AA: 16892673130
RT: 15.20
AA: 39515371599
RT: 22.54
AA: 8175914719
RT: 11.99
AA: 13695280125
RT: 2.02
AA: 1441970198
RT: 25.73
AA: 1631826953
NL: 7.00E9
TIC F: FTMS + p ESI Full ms
[300.00-2000.00] MS ICIS
RT: 17.20
AA: 71969702
NL: 1.42E7
m/z= 1304.8580-1304.8840 F:
FTMS + p ESI Full ms
[300.00-2000.00] MS ICIS
Lentinulin A
RT: 11.61
AA: 516281113
NL: 3.71E7
m/z= 1322.8683-1322.8947 F:
FTMS + p ESI Full ms
[300.00-2000.00] MS ICIS
Linear peptide +9Me
RT: 10.81
AA: 62410402
NL: 3.99E6
m/z= 1039.6816-1039.7024 F:
FTMS + p ESI Full ms
[300.00-2000.00] MS ICIS
Short linear peptide +7Me
Relative Abundance
Time (min)
0
5
10
15
20
25
30
100
80
60
40
20
0

C

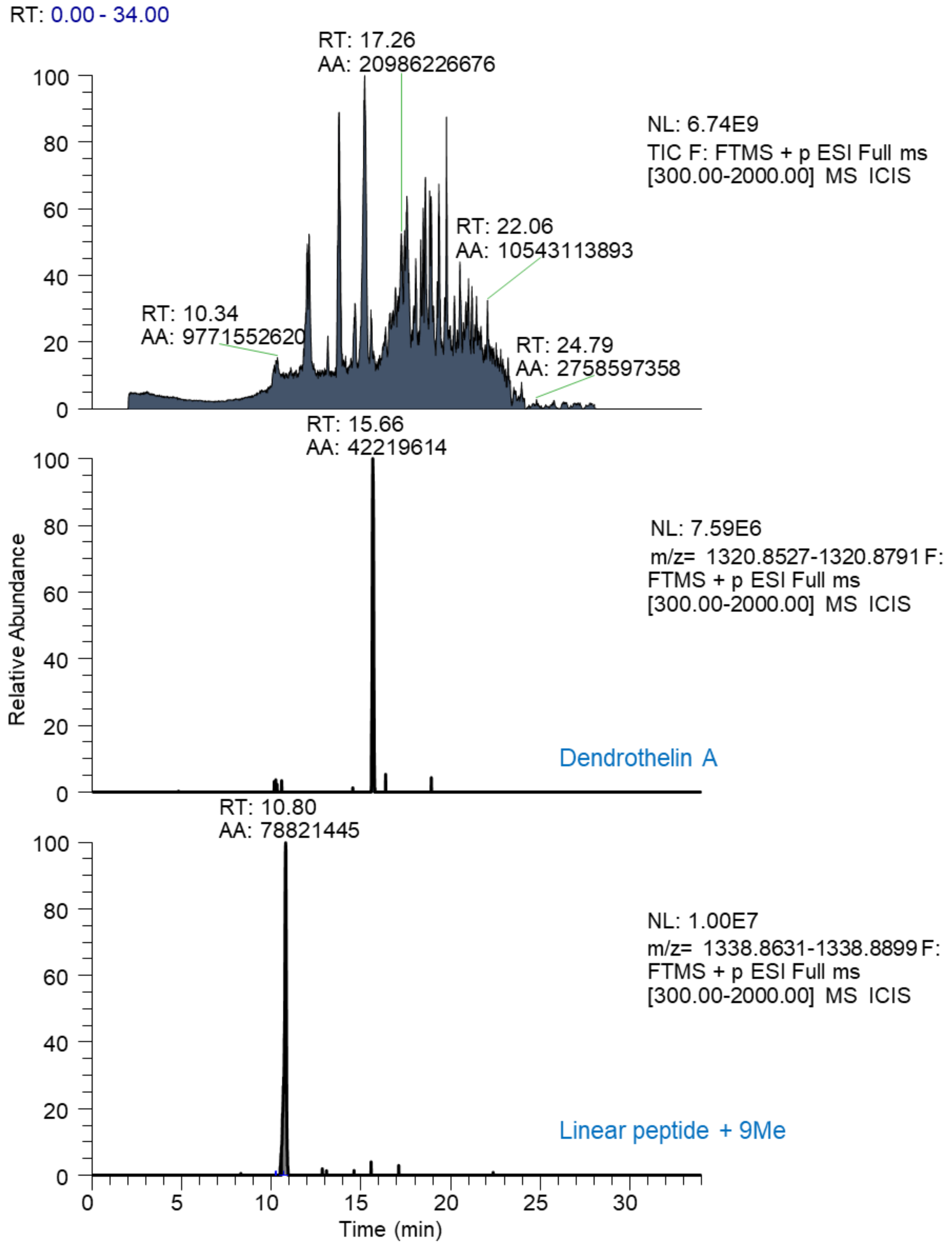

Supplementary Figure S3. Production of omphalotin A, lentinulin A and dendrothelin A in *P. pastoris*. Panels A to C depict the total ion chromatograms (TIC; top) and extracted ion chromatograms (EIC; below TIC) with the retention times (RT) and the peak areas (AA) of the various peptide species detected in the double transformants GS115-OphMA-OphP, GS115-OphMA_LedCORE-OphP, and GS115-OphMA_DbiCORE-OphP, respectively. The normalized intensities (NL) of the peptide species are also indicated.

A

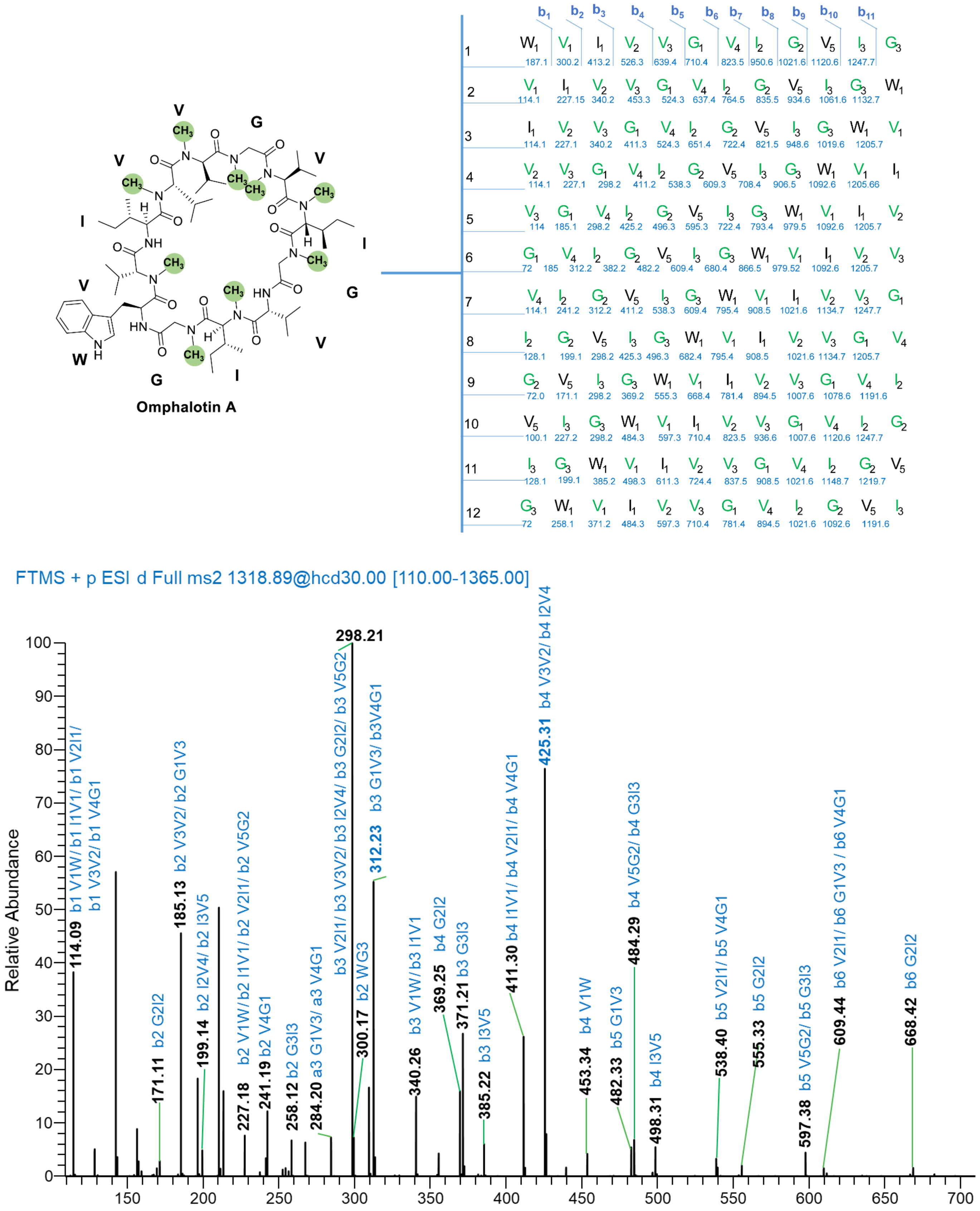
Omphalotin A
FTMS + p ESI d Full ms2 1318.89@hcd30.00 [110.00-1365.00]
Relative Abundance
298.21
425.31
312.23
114.09
185.13
484.29

B

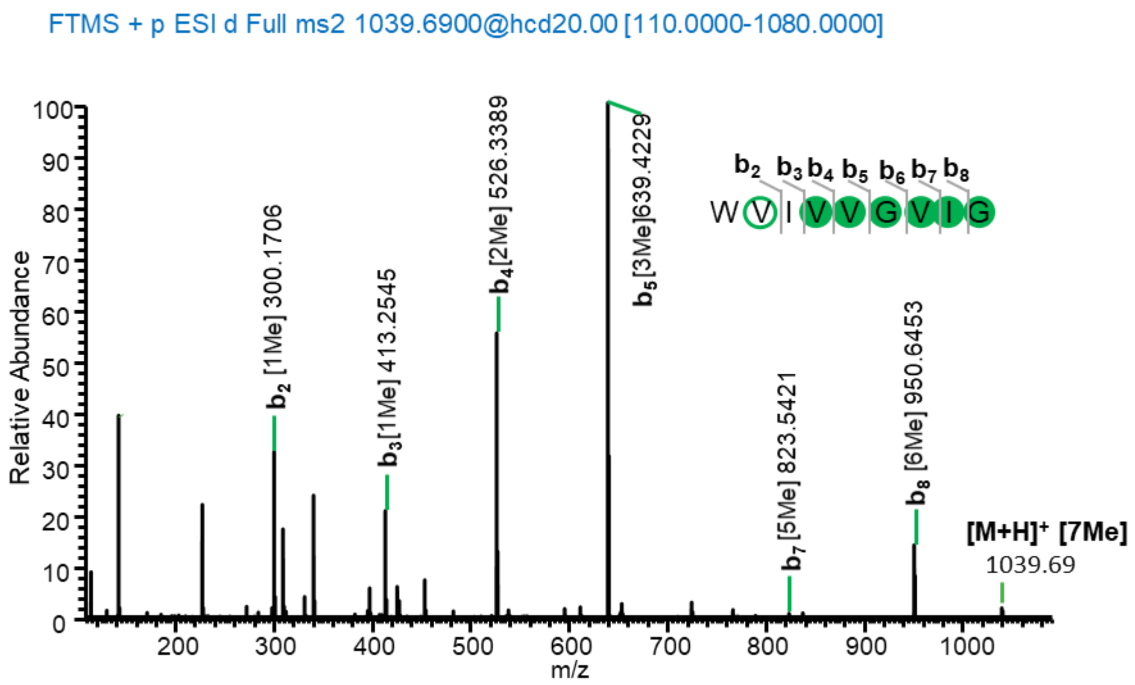

C

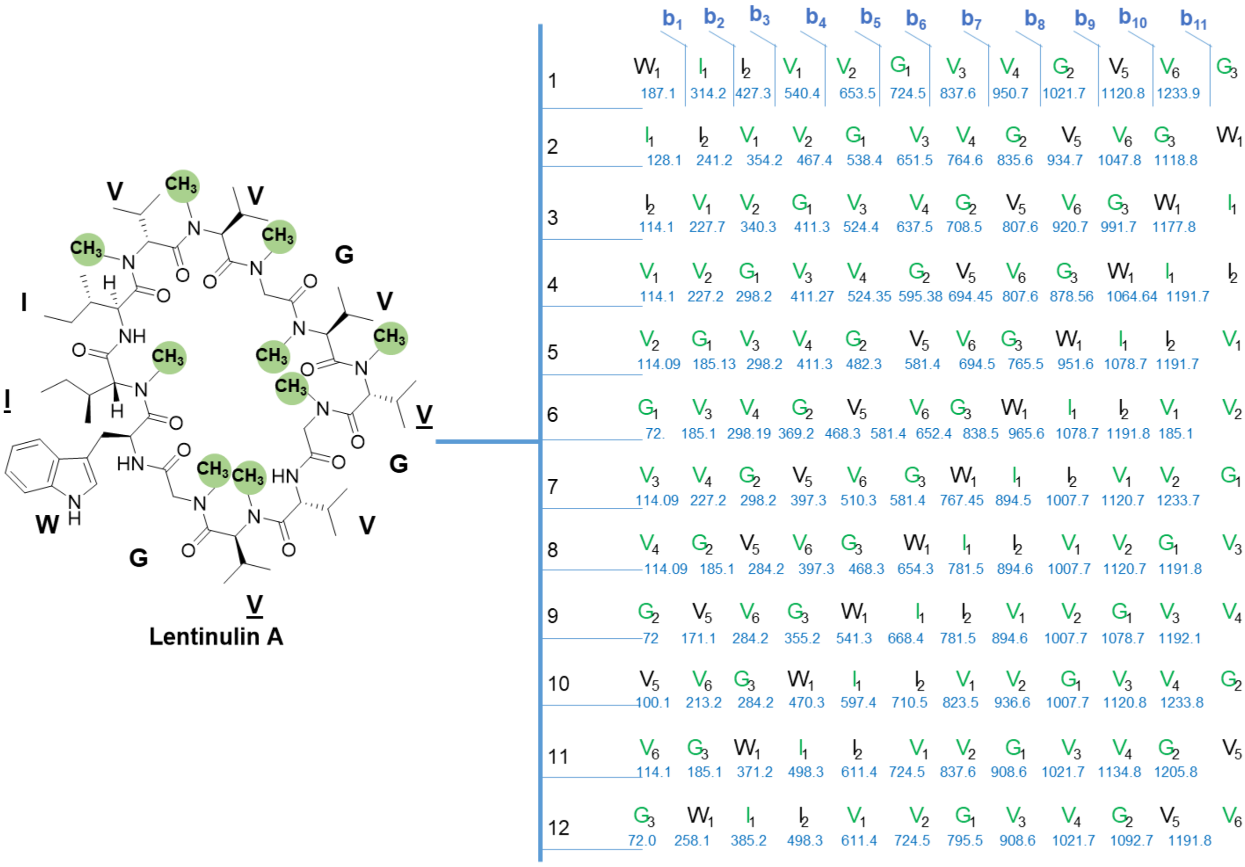

Lentinulin A

	b_1	b_2	b_3	b_4	b_5	b_6	b_7	b_8	b_9	b_{10}	b_{11}	
1	W_1 187.1	I_1 314.2	I_2 427.3	V_1 540.4	V_2 653.5	G_1 724.5	V_3 837.6	V_4 950.7	G_2 1021.7	V_5 1120.8	V_6 1233.9	G_3
2	I_1 128.1	I_2 241.2	V_1 354.2	V_2 467.4	G_1 538.4	V_3 651.5	V_4 764.6	G_2 835.6	V_5 934.7	V_6 1047.8	G_3 1118.8	W_1
3	I_2 114.1	V_1 227.7	V_2 340.3	G_1 411.3	V_3 524.4	V_4 637.5	G_2 708.5	V_5 807.6	V_6 920.7	G_3 991.7	W_1 1177.8	I_1
4	V_1 114.1	V_2 227.2	G_1 298.2	V_3 411.27	V_4 524.35	G_2 595.38	V_5 694.45	V_6 807.6	G_3 878.56	W_1 1064.64	I_1 1191.7	I_2
5	V_2 114.09	G_1 185.13	V_3 298.2	V_4 411.3	G_2 482.3	V_5 581.4	V_6 694.5	G_3 765.5	W_1 951.6	I_1 1078.7	I_2 1191.7	V_1
6	G_1 72.	V_3 185.1	V_4 298.19	G_2 369.2	V_5 468.3	V_6 581.4	G_3 652.4	W_1 838.5	I_1 965.6	I_2 1078.7	V_1 1191.8 185.1	V_2
7	V_3 114.09	V_4 227.2	G_2 298.2	V_5 397.3	V_6 510.3	G_3 581.4	W_1 767.45	I_1 894.5	I_2 1007.7	V_1 1120.7	V_2 1233.7	G_1
8	V_4 114.09	G_2 185.1	V_5 284.2	V_6 397.3	G_3 468.3	W_1 654.3	I_1 781.5	I_2 894.6	V_1 1007.7	V_2 1120.7	G_1 1191.8	V_3
9	G_2 72	V_5 171.1	V_6 284.2	G_3 355.2	W_1 541.3	I_1 668.4	I_2 781.5	V_1 894.6	V_2 1007.7	G_1 1078.7	V_3 1192.1	V_4
10	V_5 100.1	V_6 213.2	G_3 284.2	W_1 470.3	I_1 597.4	I_2 710.5	V_1 823.5	V_2 936.6	G_1 1007.7	V_3 1120.8	V_4 1233.8	G_2
11	V_6 114.1	G_3 185.1	W_1 371.2	I_1 498.3	I_2 611.4	V_1 724.5	V_2 837.6	G_1 908.6	V_3 1021.7	V_4 1134.8	G_2 1205.8	V_5
12	G_3 72.0	W_1 258.1	I_1 385.2	I_2 498.3	V_1 611.4	V_2 724.5	G_1 795.5	V_3 908.6	V_4 1021.7	G_2 1092.7	V_5 1191.8	V_6

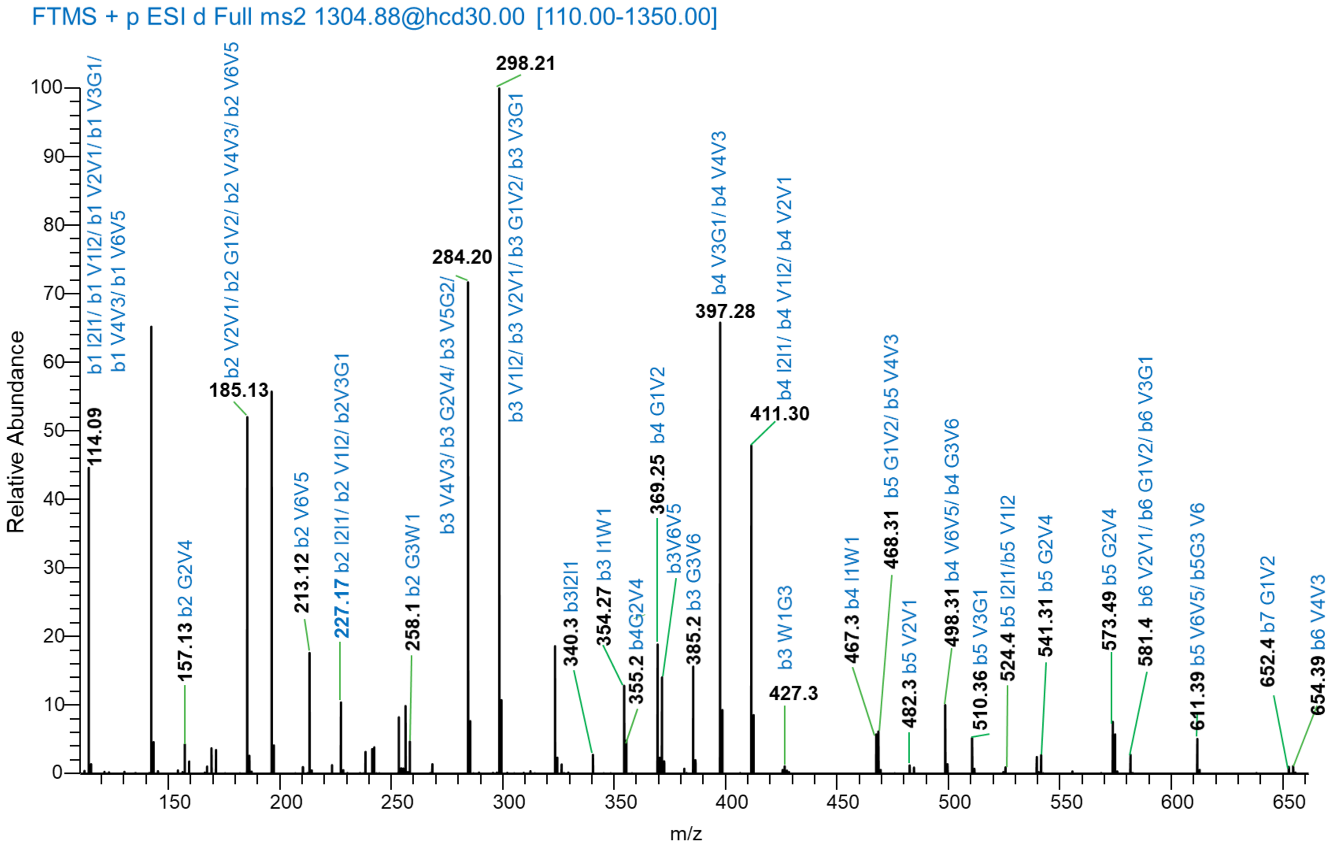
FTMS + p ESI d Full ms2 1304.88@hcd30.00 [110.00-1350.00]
Relative Abundance
m/z
114.09
b1 I2I1/ b1 V1I2/ b1 V2V1/ b1 V3G1/ b1 V4V3/ b1 V6V5
157.13 b2 G2V4
185.13
b2 V2V1/ b2 G1V2/ b2 V4V3/ b2 V6V5
213.12 b2 V6V5
227.17 b2 I2I1/ b2 V1I2/ b2V3G1
258.1 b2 G3W1
284.20
b3 V4V3/ b3 G2V4/ b3 V5G2/
298.21
b3 V1I2/ b3 V2V1/ b3 G1V2/ b3 V3G1
340.3 b3I2I1
354.27 b3 I1W1
355.2 b4G2V4
369.25 b4 G1V2
b3V6V5
385.2 b3 G3V6
397.28
b4 V3G1/ b4 V4V3
411.30
b4 I2I1/ b4 V1I2/ b4 V2V1
427.3
b3 W1G3
467.3 b4 I1W1
468.31 b5 G1V2/ b5 V4V3
482.3 b5 V2V1
498.31 b4 V6V5/ b4 G3V6
510.36 b5 V3G1
524.4 b5 I2I1/b5 V1I2
541.31 b5 G2V4
573.49 b5 G2V4
581.4 b6 V2V1/ b6 G1V2/ b6 V3G1
611.39 b5 V6V5/ b5G3 V6
652.4 b7 G1V2
654.39 b6 V4V3

D

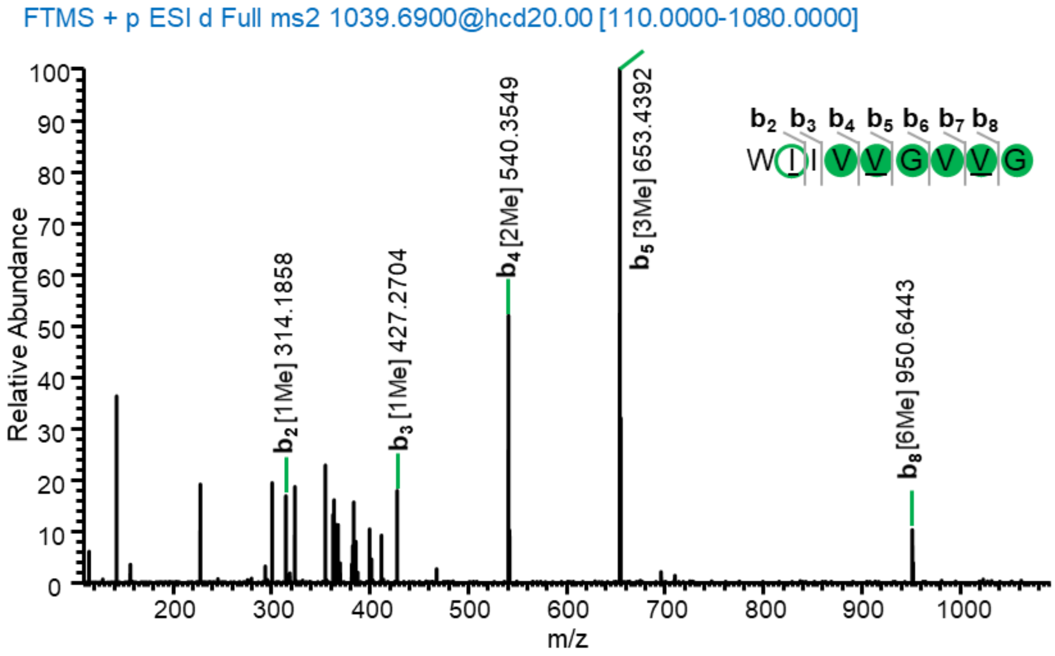
FTMS + p ESI d Full ms2 1039.6900@hcd20.00 [110.0000-1080.0000]
Relative Abundance
m/z
b2 [1Me] 314.1858
b3 [1Me] 427.2704
b4 [2Me] 540.3549
b5 [3Me] 653.4392
b8 [6Me] 950.6443
b2 b3 b4 b5 b6 b7 b8
W I I V V G V V G

E

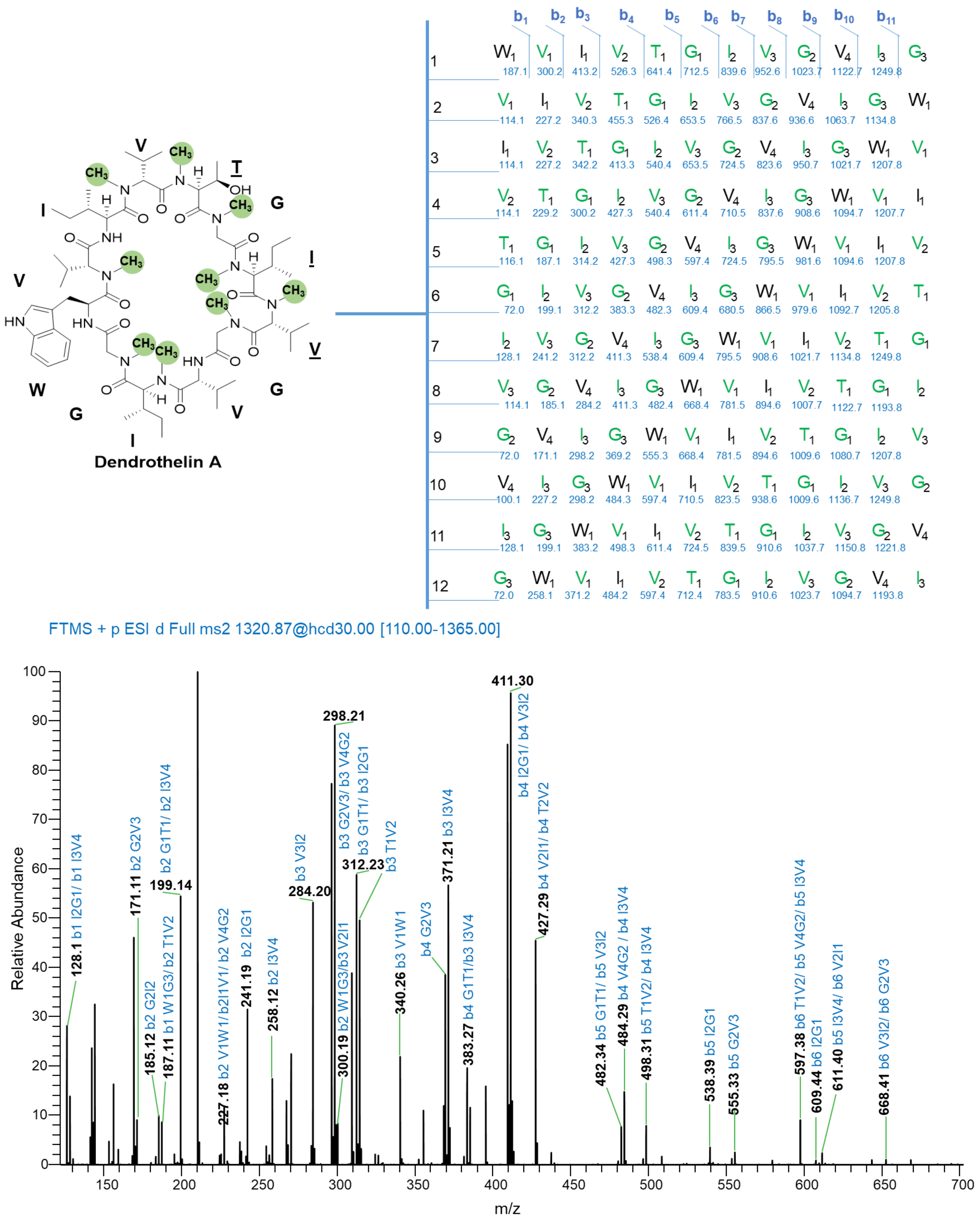

Supplementary Figure S4. MS/MS spectral annotation of peptides produced in *P. pastoris*. (A) Omphalotin A, (B) short linear peptide of omphalotin, (C) lentinulin A, (D) short linear peptide of lentinulin and (E) dendrothelin A. LC-MS/MS was performed as described in the Material and Methods section. The macrocyclic peptides can undergo ring opening at each amide bond. For this reason, 12 linear peptide fragments can be formed, yielding a total 132 possible b-ions for each macrocyclic peptide. For simplification, part of the spectrum and only the b-ions are shown. Signature

fragment masses are indicated for each b-ion detected[4]. In the peptide structure, residues different from omphalotin A are underlined. Backbone N-methylated residues are written in green.

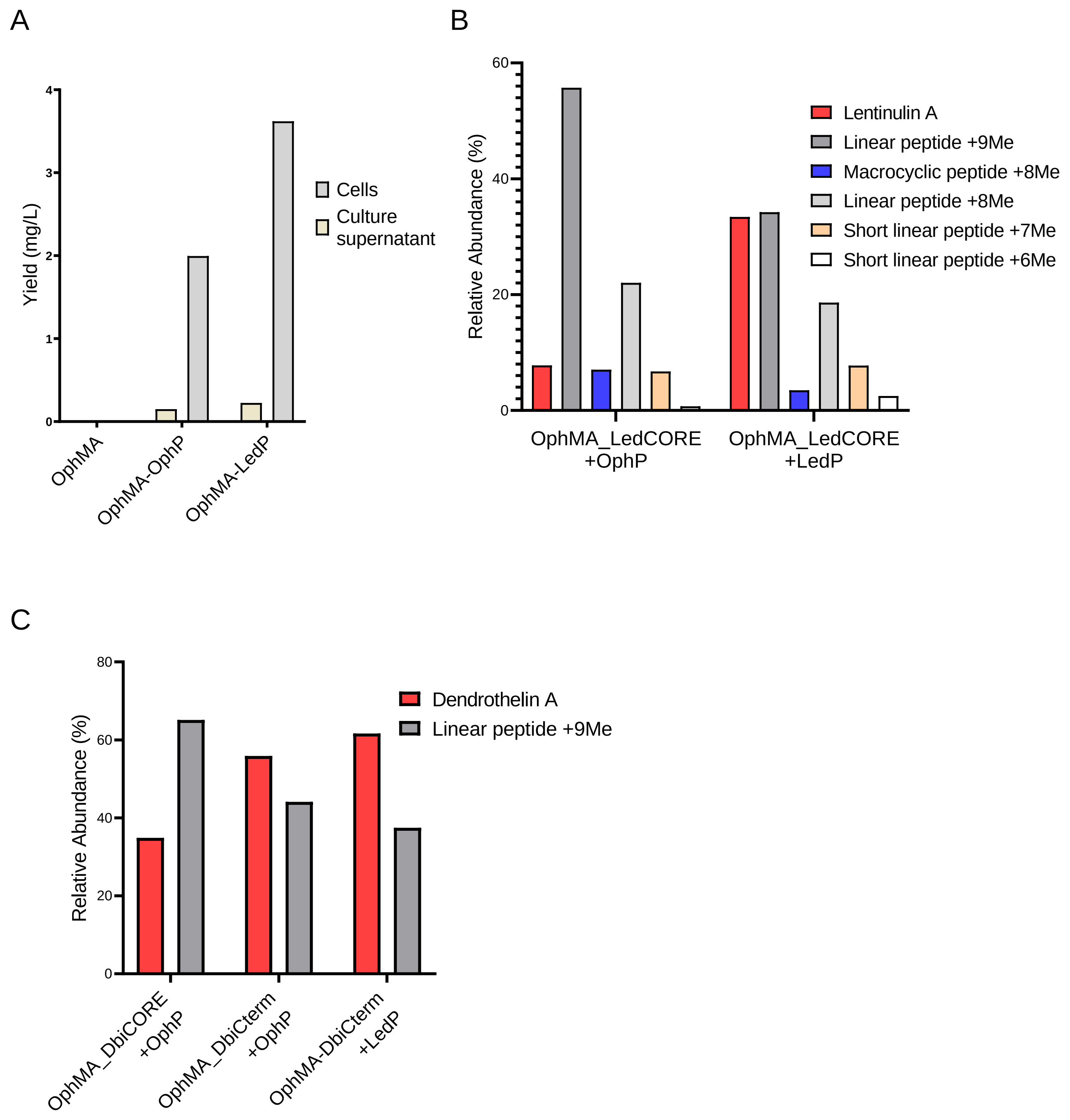

Supplementary Figure S5. Cross-reactivity of OphP homologues from different fungi in *P. pastoris*. (A) Comparison of omphalotin A production in *P. pastoris* using OphP and its homologous prolyloligopeptidase LedP from *L. edodes* using GS115-OphMA-OphP and GS115-OphMA-LedP cells, respectively. The integrated EIC peak area was compared to the one of chemically synthesized omphalotin A (216 ng/ml) which results in an estimated yield of 2 mg and 3.6 mg omphalotin A per liter culture of GS115-OphMA-OphP and GS115-OphMA-LedP cells, respectively. The relative amounts of omphalotin A identified in the cells and the culture supernatants are indicated. (B-C) Relative abundance of the different lentinulin A- and dendrothelin A-related peptides extracted from *P. pastoris* cells expressing *ophP* or ledP with different OphMA hybrids. Abundance is based on the respective integrated EIC peak areas (see Supplementary Figure S4) and the sum of the areas of all displayed species was set to 100%.

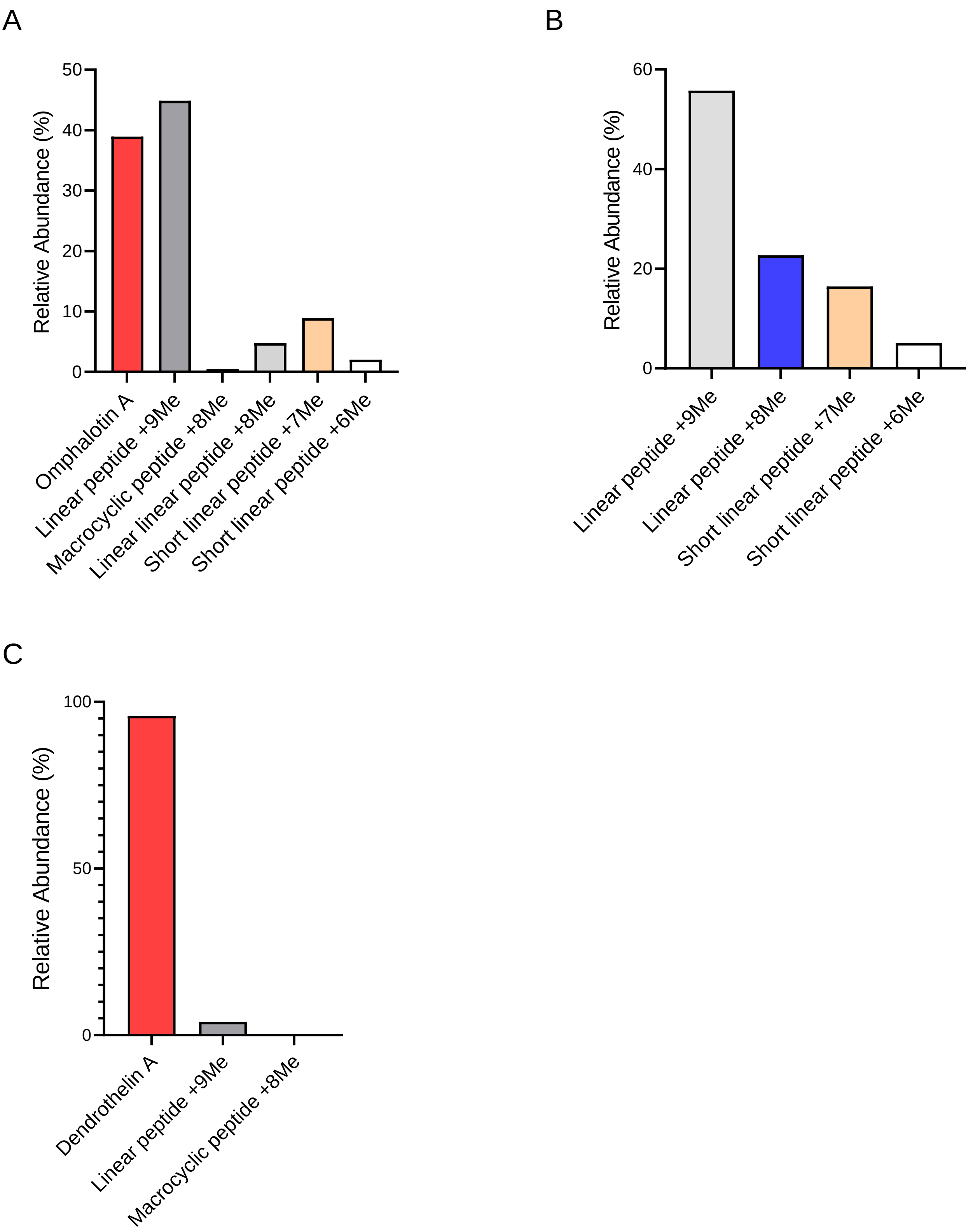

Supplementary Figure S6. Relative abundance of peptide species extracted from cultures of the original host fungi. Peptides extracted from *O. olearius* liquid culture medium (A), *L. edodes* mycelium grown on plates (B), and *D. bispora* liquid culture medium (C). Oxidized macrocyclic peptide species were not included in the representation. Relative abundance was determined as described in Fig. S5, panels B and C.

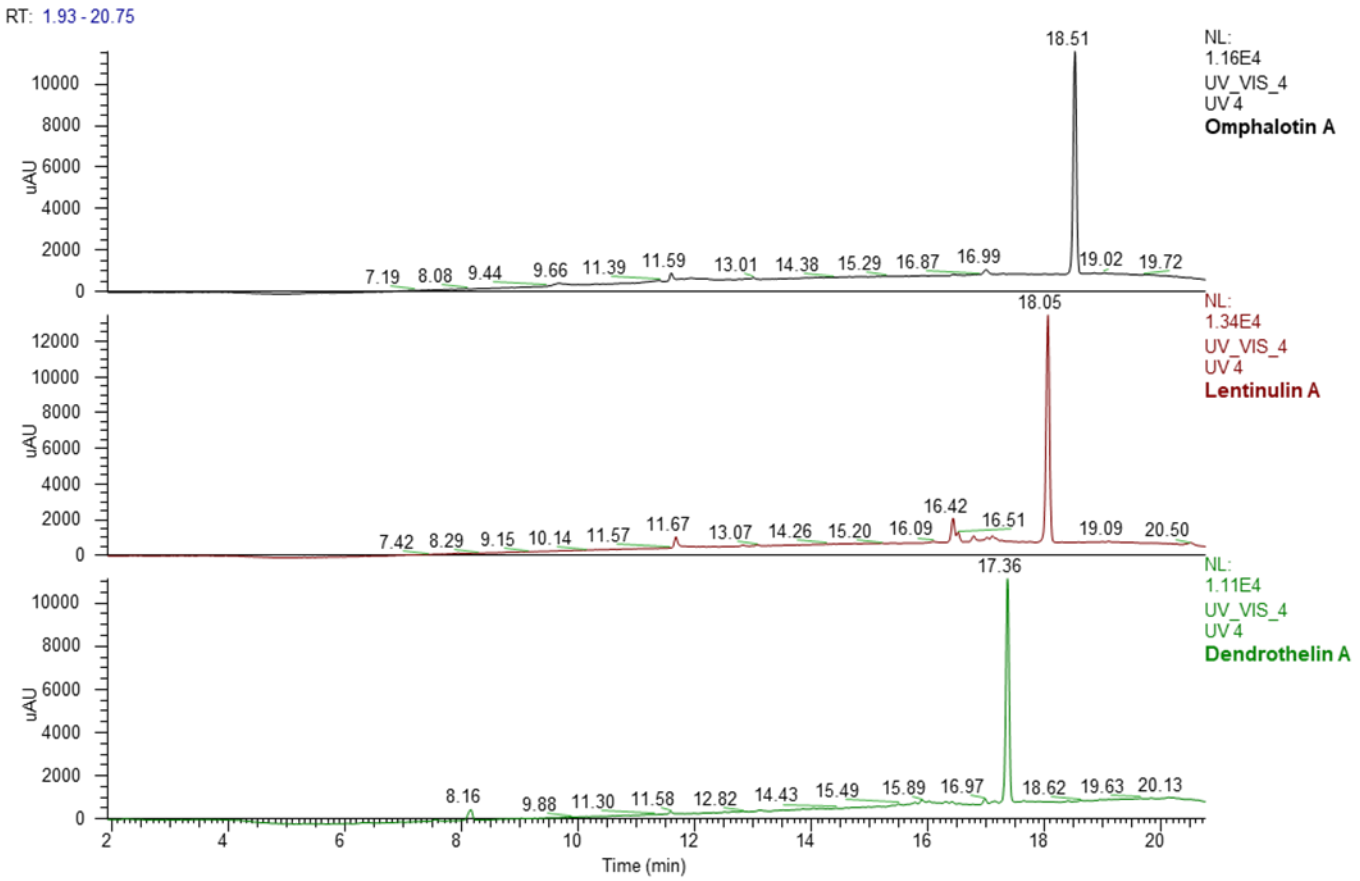

Supplementary Figure S7. HPLC chromatograms of the purified recombinant peptides. The individual peptides were extracted from 7 liters of *Pichia pastoris* culture and purified using reverse phase HPLC (see details in Materials and Methods section). Upon collection of the peptide-containing fractions, the solvent was evaporated and the dried pellets were weighed. 1200 µg, 800 µg and 400 µg of pure compounds were obtained for omphalotin A, lentinulin A and dendrothelin A, respectively. To determine the purity of the compounds, 10 µl of the concentrated samples (100 µM in methanol) was analyzed using a HPLC-MS (Thermo Scientific Q Exactive classic) system in UV visible mode (λ = 280nm). For this purpose, the LC-MS/MS method described in the Materials and Methods section was slightly modified, precisely on HPLC gradient using solvent A (water) and solvent B (acetonitrile). Briefly, the column was equilibrated with 5% B for 2 min, followed by a linear gradient up to 70% B for 13 min, and from 70% B to 100% B for 3 min, then flushing the column with 100% B for 1 min, and finally re-equilibrating the column at 5% B for 5 min. The flow rate was kept at 0.5 ml/min and the whole method took 25 min. The compound peaks were observed at 18.51 min, 18.04 min and 17.36 min for omphalotin A, lentinulin A and dendrothelin A, respectively. Based on the respective chromatograms and UV peak intensities, the purity of the recombinant peptides was estimated to be more than 95%.

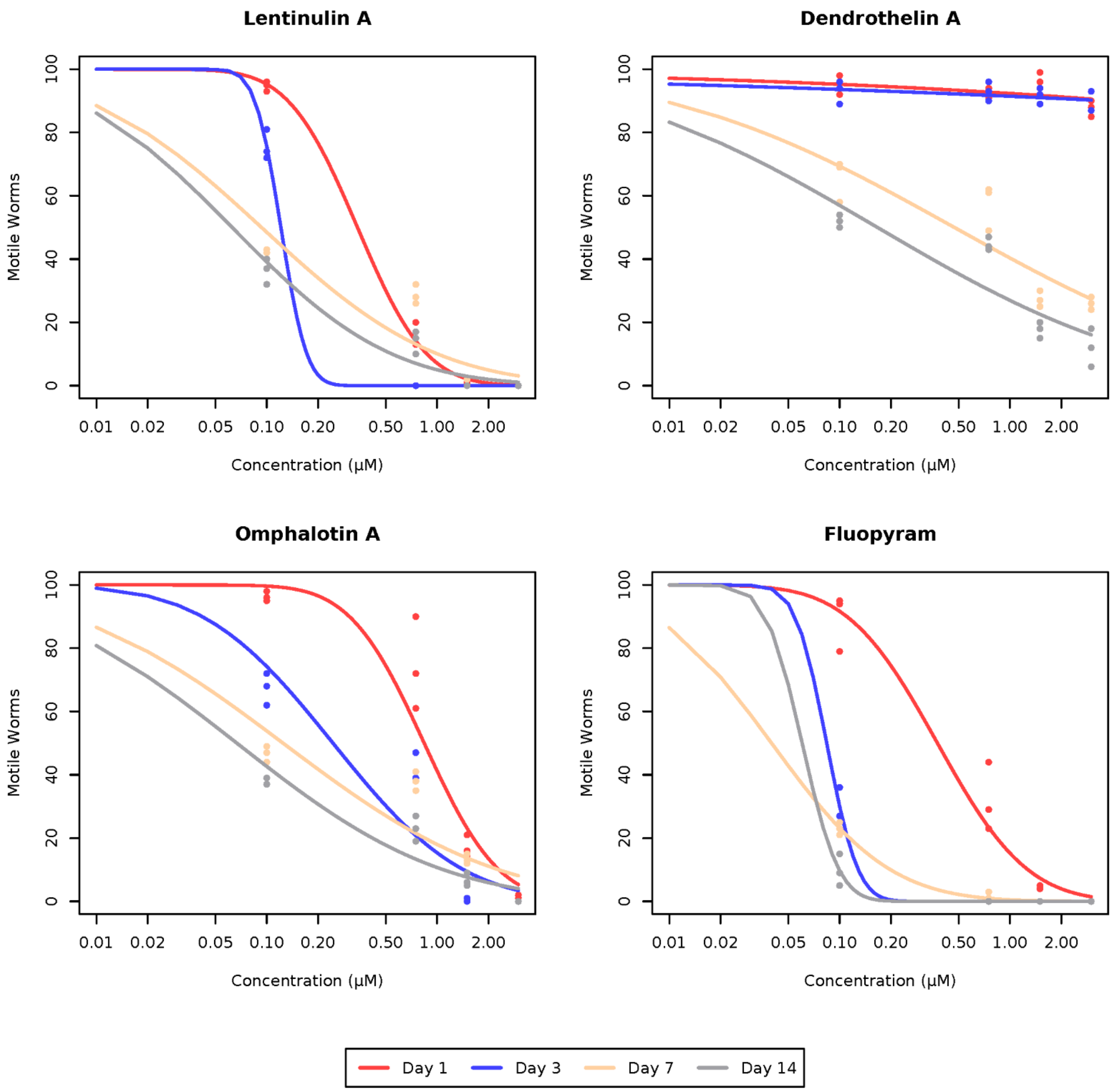

Supplementary Figure S8. Estimation of LC50 for different treatments at different time points. For each treatment at each time point, the first 100 second-stage juveniles (J2) of *M. incognita* were assessed for motility in 3 biological replicates (raw data provided as Supplementary Dataset 1). The data was imported into R 4.0.0[5] and for each data set the counts were fit by a binomial Probit model to the $\log_{10}$ of the compound molar concentration using the *glm* function. The *dose.p* function from package MASS 7.3-51.6[6] was used to estimate the lethal concentration 50% (LC50) and the standard error (SE) of this estimate, and the 95% confidence interval (CI) was calculated at LC50±1.96xSE and the values converted back from logarithmic form to molar concentration.

Supplementary Table S1. List of protein sequences. In OphMA and the hybrids thereof, the core peptide region is highlighted in bold and underlined. For StrepII-SUMOstar-OphP and StrepII-SUMOstar-LedP, SUMOstar is shown in italics and the strepII tag and TEV cleavage sites (TEVcs) are underlined.

> HisOphMA

MEHHHHHHHHTSTQTKAGSLTIVGTGIESIGQMTLQALSYIEAAAKVFYCVIDPATEAFILTKNKNCVDLYQYYDNGKSRLNTYTQMSE
LMVREVRKGLDVVGVFYGHPGVFVNPSHRALAIAKSEGYRARMLPGVSAEDCLFADLCIDPSNPGCLTYEASDFLIRDRPVSIHSHLVLF
QVGCVGIADFNFTGFDNNKFGVLVDRLEQEYGAEHPVVHYIAAMMPHQDPVTDKYTVAQLREPEIAKRVGGVSTFYIPPKARKASNL
DIIRRLELLPAGQVPDKKARIYPANQWEPDVPEVEPYRPSDQAAIAQLADHAPPEQYQPLATSKAMSDVMTKLALDPKALADYKADH
RAFAQSVPDLTPQERAALELGDSWAIRCAMKNMPSSLLDAARESGEEASQNGFPWVIVVGVIGVIGSVMSTE*

> HisOphMA-DbiCterm

MEHHHHHHHHTSTQTKAGSLTIVGTGIESIGQMTLQALSYIEAAAKVFYCVIDPATEAFILTKNKNCVDLYQYYDNGKSRLNTYTQMSE
LMVREVRKGLDVVGVFYGHPGVFVNPSHRALAIAKSEGYRARMLPGVSAEDCLFADLCIDPSNPGCLTYEASDFLIRDRPVSIHSHLVLF
QVGCVGIADFNFTGFDNNKFGVLVDRLEQEYGAEHPVVHYIAAMMPHQDPVTDKYTVAQLREPEIAKRVGGVSTFYIPPKARKASNL
DIIRRLELLPAGQVPDKKARIYPANQWEPDVPEVEPYRPSDQAAIAQLADHAPPEQYQPLATSKAMSDVMTKLALDPKALADYKADH
RAFAQSVPDLTPQERAALELGDSWAIRCAMKNMPSSLLDAARESGEEASQNGFPWVIVTGIVGVIGSVVSSA*

> HisOphMA-DbiCORE

MEHHHHHHHHTSTQTKAGSLTIVGTGIESIGQMTLQALSYIEAAAKVFYCVIDPATEAFILTKNKNCVDLYQYYDNGKSRLNTYTQMSE
LMVREVRKGLDVVGVFYGHPGVFVNPSHRALAIAKSEGYRARMLPGVSAEDCLFADLCIDPSNPGCLTYEASDFLIRDRPVSIHSHLVLF
QVGCVGIADFNFTGFDNNKFGVLVDRLEQEYGAEHPVVHYIAAMMPHQDPVTDKYTVAQLREPEIAKRVGGVSTFYIPPKARKASNL
DIIRRLELLPAGQVPDKKARIYPANQWEPDVPEVEPYRPSDQAAIAQLADHAPPEQYQPLATSKAMSDVMTKLALDPKALADYKADH
RAFAQSVPDLTPQERAALELGDSWAIRCAMKNMPSSLLDAARESGEEASQNGFPWVIVTGIVGVIGSVMSTE*

> HisOphMA-LedCORE

MEHHHHHHHHTSTQTKAGSLTIVGTGIESIGQMTLQALSYIEAAAKVFYCVIDPATEAFILTKNKNCVDLYQYYDNGKSRLNTYTQMSE
LMVREVRKGLDVVGVFYGHPGVFVNPSHRALAIAKSEGYRARMLPGVSAEDCLFADLCIDPSNPGCLTYEASDFLIRDRPVSIHSHLVLF
QVGCVGIADFNFTGFDNNKFGVLVDRLEQEYGAEHPVVHYIAAMMPHQDPVTDKYTVAQLREPEIAKRVGGVSTFYIPPKARKASNL
DIIRRLELLPAGQVPDKKARIYPANQWEPDVPEVEPYRPSDQAAIAQLADHAPPEQYQPLATSKAMSDVMTKLALDPKALADYKADH
RAFAQSVPDLTPQERAALELGDSWAIRCAMKNMPSSLLDAARESGEEASQNGFPWIIVVGVVGVVGSVMSTE*

>StrepII-SUMOstar-TEVcs-LedP

MGWSHPQFEKGGSDSEVNQEAKPEVKPEVKPETHINLKVSDGSSEIFFKIKKTTPLRRLMEAFAKRQGKEMDSLTFLYDGIEIQADQTP
*EDLDMEDNDIIEAHREQIGGENLYFQG*TSMSVPQWDPYPPVSRDETSAITYQSKLCGSVTVRDPYSALEVPFDDSEETKAFVHAQRKF
ARTYLDEIPDRETWLQTLKESWNYRRFTVPKRESDGYTYFEYNDGLQSQMSLRRVKVSEEDTILTESGPGGELFFDPNLLSLDGNAALT
GSMMSPCGKYWAYGVSEHGSDWMTTYVRKTSSPHMPSQEKGKDPGRMDDVIRYSRFFIVYWSSDSKGFFYSRYPPEDDEGKGNTP
AQNCMVYYHRLGEKQEKDTLVYEDPEHPFWLWALQLSPSGRYALLTASRDASHTQLAKIADIGTSDIQNGIQWLTIHDQWQARFVII
GDDDSTIYFMTNLEAKNYLVATLDIRHSEAGVKTLVAENPDALLISASILSTDKLVLVYLHNARHEIHVHDLNTGKQIRQIFDNLIGQFSLS
GRRDDNDMFVFHSGFTSPGTIYRFRLNEDSNKGTLFRAVQVPGLNLSDFTTESVFYPSKDGTPIHMFITRLKDTPVDGTAPVYIYGYGG
FALAMLPTFSVSTLLFCKIYRAMYVVPNIRGGSEFGESWHREGMLDKKQNVFDDFNAATKWLVANKYANKYNVAIRGGSNGGVLTT
ACANQAPELYRCVITIGGIIDMLRFPKFTFGALWRSEYGDPEDPEDFDFIYKYSPYHNIPSGDVVLPAMLFFTAAYDDRVSPLHSFKHVA
ALQYNFPNGPNPVLMRIDLNTGHFAGKSTQKMLEETADEYSFIGKSMGLVMCAQNEHASKQWSCVVT*

>StrepII-SUMOstar-TEVcs-OphP

MGWSHPQFEKGGSDSEVNQEAKPEVKPEVKPETHINLKVSDGSSEIFFKIKKTTPLRRLMEAFAKRQGKEMDSLTFLYDGIEIQADQTP
*EDLDMEDNDIIEAHREQIGGENLYFQG*TSMSFPGWGPYPPVERDETSAITYSSKLHGSVTVRDPYSQLEVPFEDSEETKAFVHSQRKFA
RTYLDENPDREAWLETLKKSWNYRRFSALKPESDGHYYFEYNDGLQSQLSLYRVRMGEEDTVLTESGPGGELFFNPNLLSLDGNAALT
GFVMSPCGNYWAYGVSEHGSDWMSIYVRKTSSPHLPSQERGKDPGRMNDKIRHVRFFIVSWTSDSKGFFYSRYPPEDDEGKGNAPA
MNCMVYYHRIGEDQESDVLVHEDPEHPFWISSVQLTPSGRYILFAASRDASHTQLVKIADLHENDIGTNMKWKNLHDPWEARFTIVG
DEGSKIYFMTNLKAKNYKVATFDANHPDEGLTTLIAEDPNAFLVSASIHAQDKLLLVYLRNASHEIHIRDLTTGKPLGRIFEDLLGQFMVS
GRRQDNDIFVLFSSFLSPGTVYRYTFGEEKGYRSLFRAISIPGLNLDDFMTESVFYPSKDGTSVHMFITRPKDVLLDGTSPVLQYGYGGFS
LAMLPTFSLSTLLFCKIYRAIYAIPNIRGGSEYGESWHREGMLDKKQNVFDDFNAATEWLIANKYASKDRIAIRGGSNGGVLTTACANQ
APGLYRCVITIEGIIDMLRFPKFTFGASWRSEYGDPEDPEDFDFIFKYSPYHNIPPPGDTVMPAMLFFTAAYDDRVSPLHTFKHVAALQH
NFPKGPNPCLMRIDLNSGHFAGKSTQEMLEETADEYSFIGKSMGLTMQTQGSVDSSRWSCVTV*

Supplementary Table S2. List of oligonucleotides. Recognition sites for restriction enzymes are underlined.

	Primers name	Sequence (5'-3')
1	OphMA_Fw	GAAAGAATTCGAAACGATGGAGCATCATCATCATCATCATCA
2	OphMA_Rv	GAAACGAGTGCGGCCGCTTATTCCGTGCTCATGACTGATC
3	LedMA_cDNA_Fw1	CACCATCATCACCACCACCACCACACTCCTACCTTAAACAAATCCGG)
4	LedMA_cDNA _Fw2	GGTACCACGTGGAAACGATGGAGCACCATCATCACCACC),
5	LedMA_cDNA_Rv	AGCTGGCGGCCGCTCAGGCGCTACTAACAAC
6	OphMA_DbiCterm_Rv	AGC TGG CGG CCG CTT AAG CAC TGC TCA CAA CCG ATC CGA TGA CCC CAA CGA TAC CCG TGA CGA TGA CCC ATG GGA AAC CG
7	OphMA_LedCore_Rv	GTT TGC GGC CGC TTA TTC CGT GCT CAT GAC TGA ACC AAC GAC ACC AAC GAC ACC AAC GAC GAT GAT CCA TGGGAA ACC GTT TTG GGA TG
8	OphMA_DbCore_Rv	CTT TTG CGG CCG CTT ATT CCG TGC TCA TGA CTG ATC CGA TGA CCC CAA CGA TAC CCG TGA CGA TGA CCC ATGGGA AAC CGT TTT GGG ATG-3
9	StrepSUMOstar_Fw1	ACC CGC AGT TCG AAA AAG GTG GTT CTG ACT CCG AGG TCA ACC AG
10	StrepSUMOstar_Fw2	GAA GGA TCC GAA ACT ATG GGT TGG AGC CAC CCG CAG TTC GAA AAA GG
11	LedP_Rv	ATT CGC GGC CGC TTA AGT GAC AAC ACA GGA CCA TTG CTT AGA AGC
12	OphP_Fw	CCA CCA CTA GTA TGT CGT TTC CAG GAT GGG GAC CAT
13	OphP_Rv	GCT TTG CGG CCG CTT AAA CTG TTA CGC AGG ACC AG
14	5-AOX1	GACTGGTTCCAATTGACAAGC
15	3-AOX1	GCAAATGGCATTCTGACATCC

Supplementary references

1 Coin, I., Beyermann, M. & Bienert, M. Solid-phase peptide synthesis: from standard procedures to the synthesis of difficult sequences. *Nat Protoc* **2**, 3247-3256, doi:10.1038/nprot.2007.454 (2007).

2 Gude, M., Ryf, J. & White, P. D. An accurate method for the quantitation of Fmoc-derivatized solid phase supports. *Letters in Peptide Science* **9**, 203-206, doi:Doi 10.1023/A:1024148619149 (2002).

3 Thern, B., Rudolph, J. & Jung, G. Total synthesis of the nematicidal cyclododecapeptide omphalotin A by using racemization-free triphosgene-mediated couplings in the solid phase. *Angew Chem Int Ed Engl* **41**, 2307-2309, doi:10.1002/1521-3773(20020703)41:13<2307::AID-ANIE2307>3.0.CO;2-Y (2002).

4 Liu, W. T. *et al.* Interpretation of tandem mass spectra obtained from cyclic nonribosomal peptides. *Anal Chem* **81**, 4200-4209, doi:10.1021/ac900114t (2009).

5 R: A language and environment for statistical computing (R Foundation for Statistical Computing, Vienna, Austria, 2020).

6 Venables, W. N. & Ripley, B. D. *Modern Applied Statistics with S*. Fourth edn, (Springer, New York, 2002).
